# TMEM72 Inhibits the proliferation by promoting cellular senescence through the activation of the P38/MAPK signaling pathway in renal cell carcinoma

**DOI:** 10.1016/j.tranon.2026.102789

**Published:** 2026-05-02

**Authors:** Fang Dai, Huming Wang, Wenhao Chu, Yu Lin, Jinqing He, Hantao Wen, Xiaotong Feng, Xudong Liu, Zihan Xu, Liangkuan Bi, Zhaojie Lyu

**Affiliations:** a Department of Urology, Peking University Shenzhen Hospital, The First Affiliated Hospital of Anhui Medical University, PKU-Shenzhen Clinical Institute of Anhui Medical University, Shenzhen, 518036, China; b Shenzhen Clinical Research Center for Urology and Nephrology, Shenzhen Key Laboratory of Male Reproduction and Genetics, Peking University Shenzhen Hospital, Shenzhen, 518036, China; c Institute of Precision Medicine, Peking University Shenzhen Hospital, PKU-Shenzhen Clinical Institute of Shantou University Medical College, Shenzhen 518036, China

**Keywords:** TMEM72, Renal cell carcinoma, MAPK signaling, Cellular senescence, Tumor microenvironment

## Abstract

**Background:**

Renal cell carcinoma (RCC) is a highly heterogeneous malignancy lacking reliable prognostic biomarkers and effective therapeutic targets. TMEM72, a kidney-enriched protein, is dysregulated in RCC, yet its biological function and underlying mechanisms remain unclear.

**Methods:**

Proteomic profiling of paired RCC and adjacent tissues was performed to identify differentially expressed proteins. TMEM72 expression and clinical relevance were validated using the TCGA-KIRC cohort and an independent patient cohort. Functional assays, including proliferation, cell cycle, and senescence analyses, were conducted in gain- and loss-of-function RCC models, and *in vivo* effects were evaluated using a xenograft model. RNA sequencing, single-cell analysis, and immune infiltration analysis were performed to explore underlying mechanisms.

**Results:**

TMEM72 was significantly downregulated in RCC and its low expression was associated with poor prognosis. TMEM72 overexpression suppressed RCC cell proliferation and tumor growth, whereas its silencing promoted tumor progression. Mechanistically, TMEM72 induced G1/S cell cycle arrest and promoted cellular senescence. Further analyses revealed that TMEM72 activated the p38/MAPK signaling pathway, leading to enhanced phosphorylation of p38 and p53, while pharmacological inhibition of p38 partially reversed these effects. Immune microenvironment analysis showed that TMEM72 was predominantly expressed in epithelial and malignant cells and was positively associated with infiltration of anti-tumor immune cells, including M1 macrophages, monocytes, and NK cells, but negatively correlated with immunosuppressive populations such as regulatory T cells and M0 macrophages.

**Conclusions:**

TMEM72 suppresses RCC progression by promoting p38/MAPK-dependent cellular senescence and may contribute to tumor immune microenvironment remodeling, highlighting its potential as a prognostic biomarker and therapeutic target.

## Highlights

• TMEM72 is downregulated in renal cell carcinoma (RCC) and correlates with poor clinical prognosis.
• TMEM72 suppresses RCC growth *in vitro* and *in vivo* by inducing cellular senescence and cell cycle arrest.
• The tumor-suppressive effect of TMEM72 is mediated through activation of the p38/MAPK signaling pathway.

## Graphical abstract

**Figure:**
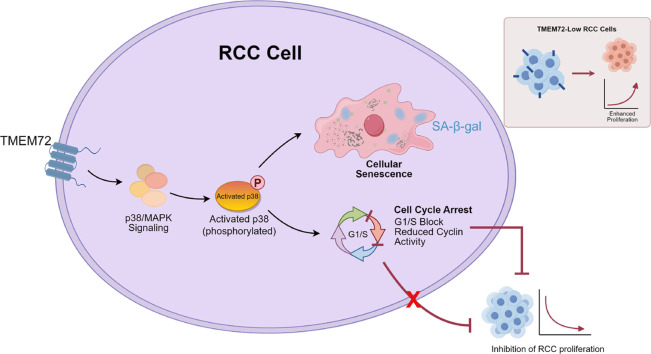

## Introduction

Among genitourinary malignancies, renal cell carcinoma (RCC) represents a major clinical burden, with a global incidence surpassed only by prostate and bladder carcinomas [1,2]. Notably, RCC accounts for the highest mortality rate among malignancies of the urinary system, with >140,000 deaths reported annually worldwide [2]. As the dominant histological subtype of kidney cancer, RCC comprises approximately 90% of all renal malignancies [3]. Although most RCC cases are diagnosed at a localized stage and are amenable to surgical resection, nearly 17% of patients present with metastatic disease at diagnosis [4]. This advanced disease stage poses a severe threat to patient survival and imposes a considerable burden on healthcare systems. Thus, unraveling the mechanistic basis of renal tumorigenesis is fundamental to advancing diagnostic tools and individualizing treatment regimens.

Transmembrane proteins (TMEMs) are ubiquitously expressed across living organisms and constitute approximately 20–30% of all proteins encoded by eukaryotic genomes [5]. Members of the TMEM family are characterized by one or multiple transmembrane domains that facilitate their integration into the lipid bilayer of cellular membranes [6]. Functionally, TMEM proteins participate in a wide range of cellular processes, including ion transport, signal transduction, molecular trafficking, and enzymatic regulation [7]. Increasing evidence has demonstrated that dysregulation of specific TMEM proteins is closely associated with tumorigenesis and patient prognosis, highlighting their potential value as diagnostic and prognostic biomarkers [7]. Evidence suggests that TMEM16A acts as a tumor-suppressive driver in prostate cancer by triggering TNF-α-dependent apoptotic pathways [8]. In colorectal cancer, knockdown of TMEM97 significantly suppresses tumor cell migration and invasion *in vitro* while inhibiting tumor growth and promoting apoptosis *in vivo* [9]. Similarly, TMEM176A, which is frequently methylated in hepatocellular carcinoma, exerts tumor-suppressive effects by restraining cell migration and invasion and by inducing apoptotic cell death [10]. The tumor immune microenvironment (TIME) is a critical determinant of tumor progression and therapeutic response, shaped not only by intrinsic tumor signaling but also by tumor-driven modulation of immune cell recruitment and function [11,12]. Cellular senescence, characterized by irreversible cell cycle arrest, acts as a fundamental tumor-suppressive mechanism [13,14]. Beyond growth inhibition, senescent cells secrete senescence-associated secretory phenotype (SASP) factors that actively remodel the immune microenvironment [15,16]. Thus, cellular senescence serves as a key link between tumor-intrinsic programs and immune regulation.

TMEM72 is a kidney-enriched transmembrane protein encoded on chromosome 10 and is predominantly expressed in renal tissue, although it can also be detected in other organs [17]. Emerging evidence suggests that TMEM72 may be involved in glutathione metabolism, a critical antioxidant pathway that maintains cellular redox homeostasis [18]. Additionally, TMEM72 is associated with the regulation of regulatory T cells (Tregs), an immune cell population with a crucial role in the tumor immune microenvironment [19]. Despite these preliminary observations, the biological functions and molecular mechanisms of TMEM72 in the context of RCC remain largely unexplored.

In the present study, we systematically investigated TMEM72 expression in RCC and evaluated its clinical relevance. Our results demonstrate that elevated TMEM72 expression is significantly associated with favorable clinical outcomes and reduced tumor cell proliferation. Mechanistically, we reveal that TMEM72 suppresses RCC progression by inducing cellular senescence, a stable growth-arrest program that acts as an intrinsic tumor-suppressive mechanism. In summary, our findings reveal that TMEM72 suppresses RCC progression and underscore its potential translational value as a prognostic biomarker and therapeutic target linked to cellular senescence.

## Materials and methods

### Clinical samples and cell lines

Human RCC tissues were obtained from patients undergoing surgical resection at Peking University Shenzhen Hospital (Shenzhen, China) following approval by the institutional ethics committee (No. 2023-184-1). All procedures were conducted in accordance with the Declaration of Helsinki, and written informed consent was obtained from all participants. For proteomic analysis, paired tumor and adjacent normal tissues from 10 RCC patients were subjected to label-free quantitative proteomic profiling using the Orbitrap Exploris 480 platform to characterize RCC-associated protein alterations. RCC cell lines were obtained from the Cell Bank of the Chinese Academy of Sciences (Shanghai, China), and only mycoplasma-free cells within 10 passages were used for experiments.

### Immunohistochemistry (IHC) staining

IHC was carried out using standard methods [20]. Briefly, paraffin-embedded sections were processed for antigen retrieval and incubated with primary antibodies at 4 °C overnight. Sections were then incubated with appropriate secondary antibodies, and immunoreactivity was visualized using DAB. The primary antibody used was anti-TMEM72 (1:200; Abcam, USA). Staining intensity and the percentage of positive cells were assessed in five randomly selected high-power fields (200 ×). The IHC score was calculated by multiplying staining intensity by staining area; scores ≤3 were defined as low expression, whereas scores >3 were classified as high expression.

### RNA isolation and RT-qPCR

Total RNA was extracted from RCC cell lines using TRIzol® reagent, followed by reverse transcription into cDNA in accordance with the manufacturer’s protocol [20]. Quantitative PCR was performed using a SYBR Green–based kit on a CFX96 real-time PCR system, with β-actin used as the internal reference gene. All primers were synthesized by Novabio. The primer sequences were as follows: β-actin (forward, 5′-CCCTGGAGAAGAGCTACGAG-3′; reverse, 5′-GGAAGGAAGGCTGGAAGAGT-3′) and TMEM72 (forward, 5′-CGTGGGCACTGAGACCTTC-3′; reverse, 5′-CTGAGCCACAAAGTAGGCCC-3′).

### Western blot assay

RCC cells were lysed in RIPA buffer on ice, and total protein levels were measured by BCA assay for subsequent western blot analysis [20]. Equal amounts of protein were separated by SDS–PAGE, transferred onto PVDF membranes, and blocked with 5% non-fat milk. Membranes were incubated with primary antibodies against TMEM72 (1:1000, Abcam, USA), E-cadherin (1:1000, Abcam), p38 (1:1000, Proteintech), p-p38 (1:1000, Abcam), p53 (1:1000, Proteintech), p-p53 (1:1000, Abcam), Lamin B1 (1:1000, CST), p21 (1:1000, Abcam), β-actin (1:1000, Abcam), MLK3 (1:2000, CST), p-MLK3 (1:2000, Abcam), TAK1 (1:1000, Proteintech), p-TAK1 (1:1000, Proteintech), MKK3 (1:1000, Abcam), MKK6 (1:1000, CST), and p-MKK3/MKK6 (1:1000, CST), followed by incubation with HRP-conjugated secondary antibodies. Protein bands were visualized using an enhanced chemiluminescence (ECL) detection system.

### Viral infection

The human TMEM72 coding sequence was amplified and cloned into a pHBLV-puro lentiviral vector. RCC cells were seeded into 6-well plates and infected with lentivirus to establish stable TMEM72-overexpressing cell lines. Infection efficiency was monitored by GFP fluorescence 48–72 h post-infection, and stable cells were selected using puromycin.

### Cell counting kit-8 (CCK-8) assay

RCC cells (2 × 10³ per well) were plated in 96-well plates and cell proliferation was measured using a CCK-8 assay [21]. Absorbance at 450 nm was measured after CCK-8 incubation at the indicated time points, and proliferation was expressed as fold change relative to baseline after background correction.

### Colony formation assay

Colony formation assays were performed as previously described [21]. Briefly, 1 × 10³ cells were seeded into 6-well plates and cultured for 2 weeks. Colonies were then fixed with paraformaldehyde, stained with crystal violet, and imaged.

### Flow cytometric analysis of the cell cycle

RCC cells were fixed in 75% cold ethanol and stained with PI after RNase A treatment for flow cytometric cell cycle analysis. Flow cytometric analysis was performed on a Beckman instrument to determine the proportion of cells in each cell cycle phase.

### Senescence β-Galactosidase staining kit

To assess cellular senescence, cells were subjected to SA-β-gal staining and senescent cells were identified by blue-green signals under light microscopy [22].

### Transcriptome sequencing

Following RNA quality assessment, qualified samples were used for library construction and high-throughput transcriptome sequencing performed by Sangon Biotechnology (Shanghai, China).

### *In vivo* xenograft model

All animal experiments were approved by the Animal Ethics Committee of Shenzhen PKU-HKUST Medical Center (NO. 2023–425). Twelve 4–6-week-old NOD/SCID mice were randomly assigned to the vector or TMEM72 group. ACHN cells were resuspended in PBS mixed with Matrigel and subcutaneously injected into the dorsal flank of each mouse (5 × 10⁶ cells per mouse). Tumor volume was measured weekly and calculated using the formula: (length × width²)/2. Mice were sacrificed 4 weeks after injection, and tumors were excised, weighed, photographed, and subjected to further immunohistochemical analysis.

### Multiplex immunofluorescence

Multiplex immunofluorescence staining was performed on mouse tumor tissues using a commercial kit (Servicebio, China). Paraffin-embedded sections were deparaffinized, underwent antigen retrieval, and were sequentially incubated with primary antibodies.

### Bioinformatics analysis

The single-cell RNA sequencing dataset (GSE159115) was downloaded from the TISCH database, which includes comprehensive annotations for various cell types in RCC [23,24]. Differential expression analysis between Vector and TMEM72 groups was conducted using the limma package in R (v4.0). Genes meeting the criteria of |log₂FC| > 2 and adjusted P < 0.05 were defined as differentially expressed. Shared DEGs were identified using FunRich, and expression patterns were visualized by heatmaps generated from Robust Rank Aggreg (RRA) analysis. Functional annotation was performed using the DAVID database (v6.8), including Gene Ontology (GO) enrichment across biological processes, molecular functions, and cellular components, as well as Kyoto Encyclopedia of Genes and Genomes (KEGG) pathway analysis to identify significantly enriched pathways. Using the median expression level of TMEM72 as the boundary, TCGA-KIRC patients were divided into high-expression group and low-expression group to evaluate their immunological correlation. The infiltration proportions of 22 immune cell subsets were quantified based on the CIBERSORT algorithm, and the relationship between TMEM72 expression and immune cell infiltration was explored through Spearman correlation analysis [25].

### Statistics

Statistical analyses were carried out using GraphPad Prism 8. Data are expressed as mean ± SD from at least three independent experiments. Differences between two groups were evaluated using Student’s *t*-test, while comparisons among multiple groups were assessed by one-way ANOVA. Survival analyses were performed using the Kaplan–Meier method. A two-sided P < 0.05 was considered statistically significant.

## Result

### TMEM72 is downregulated in RCC and associated with patient prognosis

**Fig. 1:**
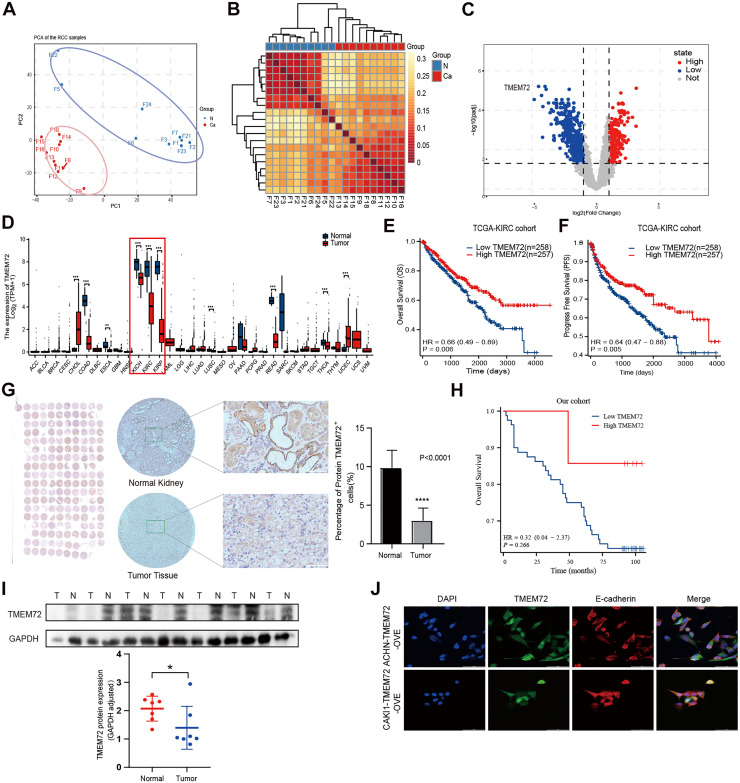
TMEM72 is downregulated in RCC and associated with patient prognosis. (A) Principal component analysis (PCA) of proteomic profiles showing clear separation between RCC tumor tissues and paired adjacent normal tissues (n = 10). (B) Heatmap of differentially expressed proteins between RCC and normal tissues (n = 10) identified by label-free quantitative proteomics. (C) Volcano plot illustrating significantly upregulated and downregulated proteins in RCC tissues, with TMEM72 highlighted among the most downregulated proteins (n = 10). (D) Pan-cancer analysis of TMEM72 expression across 33 tumor types and corresponding normal tissues based on TCGA datasets. (E) Kaplan–Meier analysis of overall survival (OS) in the TCGA-KIRC cohort stratified by TMEM72 expression (n = 515). (F) Kaplan–Meier analysis of progression-free interval (PFI) in the TCGA-KIRC cohort according to TMEM72 expression levels (n = 515). (G) Representative immunohistochemical (IHC) staining of TMEM72 in paired RCC and adjacent normal tissues from the validation cohort (n = 90). (H) Kaplan–Meier analysis of OS in the institutional validation cohort based on TMEM72 expression (n = 87). (I) Western blot analysis of TMEM72 protein levels in RCC and matched normal tissues, along with corresponding quantitative analysis (n = 7). (J) Immunofluorescence staining of TMEM72 in ACHN and Caki-1 cells with TMEM72 overexpression. TMEM72 is shown in green, E-cadherin (E-ca) in red, and nuclei (DAPI) in blue, indicating predominant membrane localization and co-localization with E-cadherin.

Paired tumor and adjacent normal tissues from 10 patients with renal cell carcinoma (RCC) were subjected to label-free quantitative proteomic analysis using the Orbitrap Exploris 480 platform to characterize RCC-associated protein alterations (Fig. 1A). Differential expression analysis revealed a distinct proteomic profile between tumor and normal tissues (Fig. 1B), among which TMEM72 was one of the most significantly downregulated proteins in RCC tissues (Fig. 1C). Consistently, TMEM72 expression was nearly absent in RCC tissues but robustly expressed in normal kidney tissues (Fig. 1D). Kaplan–Meier survival analysis based on the TCGA-KIRC cohort demonstrated that low TMEM72 expression was significantly associated with reduced overall survival (OS) (Fig. 1E). Similar results were observed for progression-free interval (PFI), indicating that low TMEM72 expression predicted poorer prognosis (Fig. 1F). Cox regression analysis in the TCGA-KIRC cohort (n = 541) showed that high TMEM72 expression was significantly associated with improved OS in univariate analysis, but only exhibited a protective trend without statistical significance in multivariate analysis. Multivariate analysis further identified distant metastasis and age > 60 years as independent adverse prognostic factors (Table 1). TMEM72 protein expression was further validated by immunohistochemistry (IHC) in 90 paired RCC and adjacent normal tissues, confirming markedly reduced expression in tumor tissues (Fig. 1G). Kaplan–Meier analysis of our institutional cohort suggested that low TMEM72 expression was associated with a trend toward poorer OS, although the difference did not reach statistical significance (Fig. 1H). A total of 90 patients were initially included, of whom 3 were lost to follow-up, leaving 87 patients for survival analysis. In this cohort, TMEM72 expression was not significantly associated with OS. Univariate analysis indicated that higher T, N, and M stages were associated with worse prognosis, whereas early clinical stage and age ≤ 60 years were associated with better survival. Multivariate analysis demonstrated that only clinical stage and age were independent prognostic factors (Table 2). Western blot analysis further confirmed that TMEM72 protein levels were significantly lower in RCC tissues compared with matched normal tissues (Fig. 1I). Finally, TMEM72-overexpressing RCC cell models were established. Immunofluorescence analysis revealed that TMEM72 was predominantly localized to the cell membrane and co-localized with the epithelial marker E-cadherin in renal cancer cells (Fig. 1J).

**Table 1:** Univariate and multivariate Cox proportional hazards regression analyses of TMEM72 expression and clinicopathological characteristics in the TCGA-KIRC cohort.

Characteristics	Total(N)	Univariate analysis		Multivariate analysis	
		Hazard ratio (95% CI)	P value	Hazard ratio (95% CI)	P value
TMEM72	541				
Low	270	Reference		Reference	
High	271	0.626 (0.463–0.846)	0.002	0.681 (0.445–1.043)	0.077
Pathologic T stage	541				
T1&T2	350	Reference		Reference	
T3&T4	191	3.210 (2.373–4.342)	< 0.001	1.483 (0.651–3.377)	0.349
Pathologic N stage	258				
N0	242	Reference		Reference	
N1	16	3.422 (1.817–6.446)	< 0.001	1.809 (0.909–3.599)	0.091
Pathologic M stage	508				
M0	429	Reference		Reference	
M1	79	4.401 (3.226–6.002)	< 0.001	2.813 (1.667–4.747)	< 0.001
Pathologic stage	538				
Stage I&Stage II	332	Reference		Reference	
Stage III&Stage IV	206	3.910 (2.852–5.360)	< 0.001	1.238 (0.493–3.111)	0.650
Gender	541				
Male	354	Reference			
Female	187	1.083 (0.796–1.473)	0.613		
Age	541				
≤ 60	269	Reference		Reference	
> 60	272	1.791 (1.319–2.432)	< 0.001	1.849 (1.195–2.861)	0.006
Histologic grade	533				
G1&G2	250	Reference		Reference	
G3&G4	283	2.665 (1.898–3.743)	< 0.001	1.578 (0.961–2.591)	0.072

**Table 2:** Univariate and multivariate Cox proportional hazards regression analyses of TMEM72 expression and clinicopathological characteristics in the our RCC cohort.

Characteristics	Total(N)	Univariate analysis		Multivariate analysis	
		Hazard ratio (95% CI)	P value	Hazard ratio (95% CI)	P value
TMEM72	87				
low	80	Reference			
high	7	0.323 (0.044–2.369)	0.266		
Pathologic T	87				
T1&T2	81	Reference		Reference	
T3	6	3.710 (1.290–10.664)	0.015	2.378 (0.523–10.811)	0.262
Pathologic N	87				
N0	85	Reference		Reference	
N1	2	14.139 (2.920–68.454)	< 0.001	4.828 (0.928–25.117)	0.061
Pathologic M	87				
M0	85	Reference		Reference	
M1	2	10.098 (2.223–45.861)	0.003	6.284 (0.777–50.821)	0.085
Stage	87				
Stage III&Stage IV	27	Reference		Reference	
Stage I&Stage II	60	0.187 (0.090–0.388)	< 0.001	0.194 (0.090–0.418)	< 0.001
Gender	86				
Female	30	Reference			
Male	56	0.887 (0.431–1.828)	0.746		
Age	85				
> 60	38	Reference		Reference	
≤ 60	47	0.332 (0.156–0.705)	0.004	0.307 (0.137–0.686)	0.004

### TMEM72 expression is associated with remodeling of the tumor immune microenvironment

**Fig. 2:**
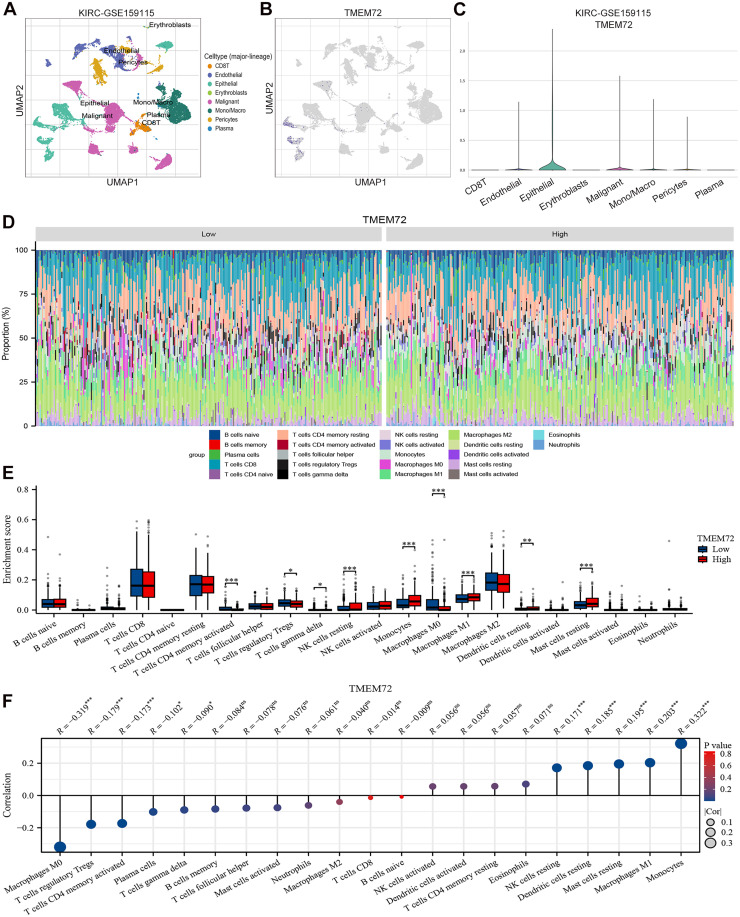
TMEM72 expression is associated with remodeling of the tumor immune microenvironment. (A) UMAP visualization of single-cell RNA-seq data (GSE159115) showing the major cell populations in the KIRC tumor microenvironment, including epithelial cells, malignant cells, fibroblasts, endothelial cells, and immune cell subsets. (B) Feature plot displaying the distribution of TMEM72 expression across different cell types, showing predominant expression in epithelial and malignant cells. (C) Violin plot showing the expression of TMEM72 in different cell types, with higher expression observed in Epithelial and Malignant cells. (D) Immune cell composition in TMEM72-high and TMEM72-low groups from the TCGA-KIRC cohort estimated by the CIBERSORT algorithm. (E) Differential analysis of tumor-infiltrating immune cells between TMEM72-high and TMEM72-low groups. (F) Correlation analysis between TMEM72 expression and the abundance of tumor-infiltrating immune cells.

To characterize the cellular distribution of TMEM72 in the tumor microenvironment of KIRC, we analyzed the single-cell RNA-seq dataset GSE159115. UMAP clustering identified major cell populations, including epithelial cells, malignant cells, fibroblasts, endothelial cells, and diverse immune subsets such as CD8⁺ T cells and macrophages (Fig. 2A). Feature plot analysis revealed that TMEM72 expression was predominantly enriched in epithelial and malignant cells, with minimal expression in stromal and immune compartments (Fig. 2B). This pattern was further confirmed by quantitative comparisons across annotated cell types (Fig. 2C). To investigate the immunological relevance of TMEM72, tumor samples from the TCGA-KIRC cohort were stratified into TMEM72-high and TMEM72-low groups, and immune cell composition was estimated using the CIBERSORT algorithm. Significant differences in immune infiltration patterns were observed between the two groups (Fig. 2D). Specifically, the TMEM72-high group exhibited increased infiltration of γδ T cells, resting NK cells, monocytes, M1 macrophages, resting dendritic cells, and resting mast cells. In contrast, the TMEM72-low group showed higher proportions of activated CD4⁺ memory T cells, regulatory T cells (Tregs), and M0 macrophages (Fig. 2E). Consistently, correlation analysis demonstrated that TMEM72 expression was positively associated with resting NK cells, resting dendritic cells, resting mast cells, M1 macrophages, and monocytes, while it was negatively correlated with M0 macrophages, Tregs, activated CD4⁺ memory T cells, plasma cells, and γδ T cells (Fig. 2F).

### Overexpressing TMEM72 inhibited the proliferation of RCC cells *in vitro*

**Fig. 3:**
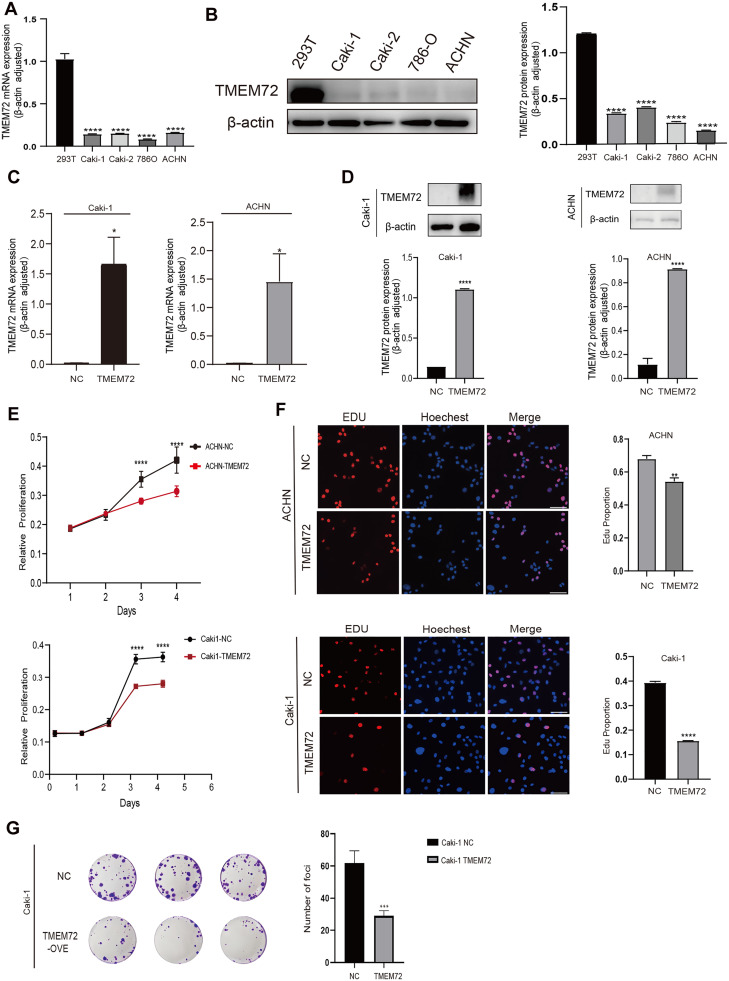
TMEM72 overexpression inhibits proliferation of RCC cells *in vitro*. (A) RT-qPCR analysis of TMEM72 mRNA expression in RCC cell lines and 293T cells. (B) Western blot analysis of TMEM72 protein levels in RCC cell lines and 293T cells, along with corresponding quantitative analysis. (C) RT-qPCR analysis confirming TMEM72 overexpression in Caki-1 and ACHN cells transfected with TMEM72 or vector control. (D) Western blot validation of TMEM72 overexpression in Caki-1 and ACHN cells, with corresponding quantitative analysis. (E) CCK-8 assay showing reduced proliferation of TMEM72-overexpressing Caki-1 and ACHN cells compared with vector controls. (F) EdU incorporation assay with fluorescence imaging (EdU, red; Hoechst, nuclear staining) and quantitative analysis, demonstrating decreased proliferation in TMEM72-overexpressing cells. (G) Representative images and quantification of colony formation assays in Caki-1 cells with TMEM72 overexpression or vector control.

Comparative analyses revealed that TMEM72 expression was significantly higher in normal renal epithelial cells than in RCC cell lines, as confirmed by quantitative PCR and western blotting (Fig. 3A–B). To further investigate the biological function of TMEM72, two RCC cell lines with low endogenous TMEM72 expression, Caki-1 and ACHN, were selected for gain-of-function studies. Cells were stably transduced with a lentiviral vector encoding full-length human TMEM72 or an empty vector control. Successful overexpression was verified at both the mRNA and protein levels by qPCR and western blotting (Fig. 3C–D). Functional assays demonstrated that TMEM72 overexpression significantly inhibited RCC cell proliferation, as evidenced by CCK-8 and EdU incorporation assays (Fig. 3E–F). In addition, colony formation assays further confirmed that TMEM72 overexpression markedly reduced the proliferative capacity of RCC cells (Fig. 3G).

### The downregulation of TMEM72 rescued the inhibited proliferation in RCC cells

**Fig. 4:**
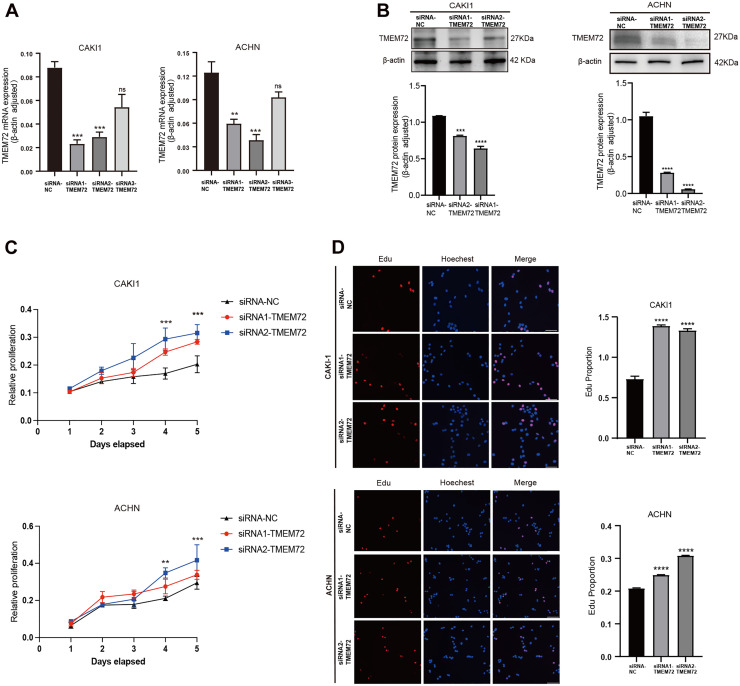
TMEM72 knockdown reverses its inhibitory effect on RCC cell proliferation *in vitro*. (A) RT-qPCR analysis of TMEM72 mRNA expression in Caki-1 and ACHN cells transfected with siRNA-NC or three independent TMEM72-targeting siRNAs. (B) Western blot analysis confirming TMEM72 protein knockdown in Caki-1 and ACHN cells, along with corresponding quantitative analysis. (C) CCK-8 assay showing that TMEM72 downregulation significantly promotes proliferation of Caki-1 and ACHN cells. EdU incorporation assay with fluorescence imaging (EdU, red; Hoechst, nuclear staining) and quantitative analysis, demonstrating that TMEM72 silencing enhances proliferative activity in TMEM72-overexpressing Caki-1 and ACHN cells compared with siRNA-NC controls.

Given the low endogenous expression of TMEM72 in RCC cells, Caki-1 and ACHN cells stably overexpressing TMEM72 were selected for siRNA-mediated knockdown studies. TMEM72 expression was robustly suppressed at both the mRNA and protein levels by two independent siRNAs (si-TMEM72#1 and si-TMEM72#2) (Fig. 4A–B). TMEM72 silencing significantly enhanced RCC cell proliferation, as evidenced by increased cell growth in CCK-8 assays (Fig. 4C) and a higher proportion of EdU-positive cells (Fig. 4D).

### TMEM72 promotes cellular senescence in RCC through activation of the MAPK signaling pathway

**Fig. 5:**
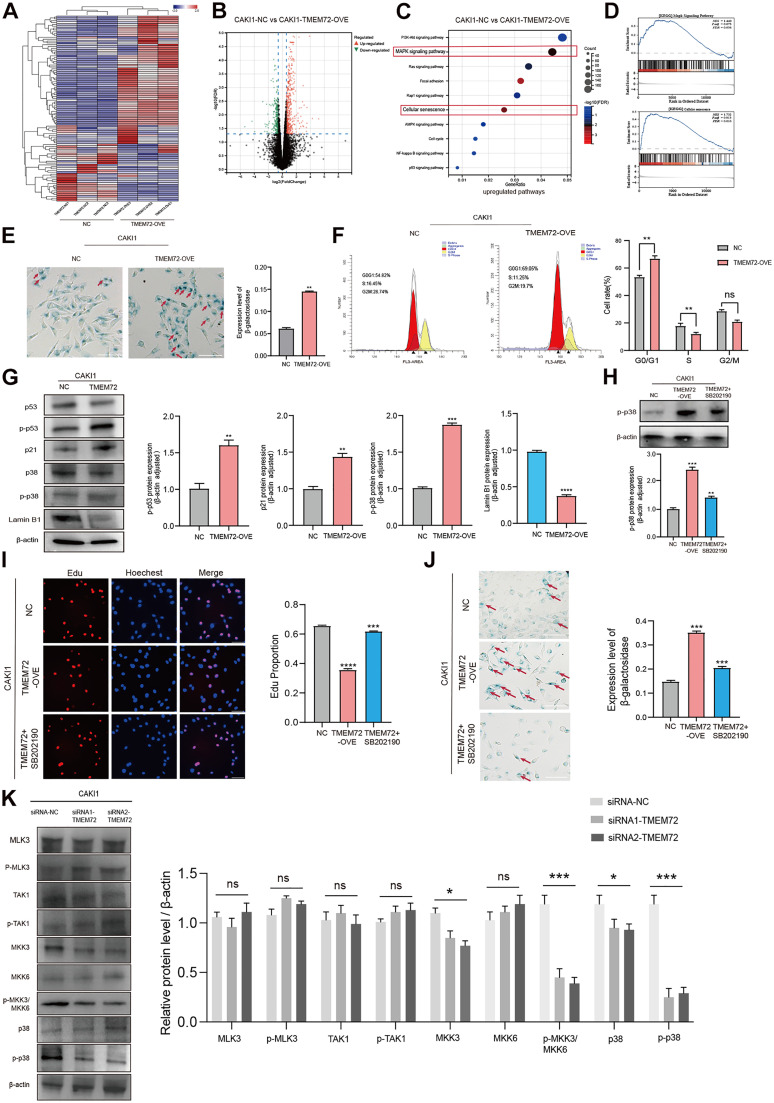
TMEM72 promotes cellular senescence in renal cell carcinoma by activating the p38/MAPK signaling pathway. (A–C) Transcriptomic analysis of RCC cells overexpressing TMEM72 and vector controls. (A) Heatmap showing differentially expressed genes in three paired TMEM72-overexpressing and control RCC cell samples (red indicates high expression, blue indicates low expression). (B) Volcano plot illustrating differentially expressed genes between TMEM72-overexpressing and control cells (red indicates upregulated genes, green indicates downregulated genes). (C) Enrichment analysis highlighting significant overrepresentation of cellular senescence–associated gene sets upon TMEM72 overexpression. (D) Gene set enrichment analysis (GSEA) demonstrating significant enrichment of the MAPK signaling pathway in TMEM72-overexpressing RCC cells. (E) Senescence-associated β-galactosidase and lysosomal β-glycosidase staining showing increased cellular senescence in TMEM72-overexpressing cells compared with vector controls. (F) Flow cytometric analysis of cell-cycle distribution revealing G1/S phase arrest in RCC cells overexpressing TMEM72. (G) Western blot analysis of senescence-associated markers, showing decreased Lamin B1 expression and increased phosphorylation of p53 (p-p53) following TMEM72 overexpression, along with corresponding quantitative analysis. (H) Western blot analysis of phosphorylated p38 MAPK (p-p38) and p-p53 in RCC cells with TMEM72 overexpression, with or without treatment with the p38 MAPK inhibitor SB202190, along with corresponding quantitative analysis. (I–J) Functional rescue experiments following p38 MAPK inhibition. (I) EdU incorporation assays (EdU, red; Hoechst, nuclear staining) showing increased proliferation upon SB202190 treatment in TMEM72-overexpressing RCC cells. (J) Senescence-associated β-galactosidase and lysosomal β-glycosidase staining showing reduced cellular senescence after SB202190 treatment. (K) Western blot analysis of MAPK pathway-related proteins in TMEM72-silenced cells, along with corresponding quantitative analysis.

Transcriptomic analysis revealed that TMEM72 overexpression was significantly enriched in gene sets associated with cellular senescence (Fig. 5A–C). Consistently, GSEA of RNA-seq data demonstrated prominent enrichment of the MAPK signaling pathway in TMEM72-overexpressing cells (Fig. 5D). Senescence-associated β-galactosidase and lysosomal β-glycosidase staining further confirmed a marked increase in senescent cells upon TMEM72 overexpression compared with vector controls (Fig. 5E). In addition, TMEM72 overexpression induced a pronounced G1/S phase arrest in RCC cells, with no significant changes observed in control cells, indicating suppression of cell-cycle progression (Fig. 5F). At the molecular level, TMEM72 overexpression induced a senescence-like phenotype, as evidenced by decreased Lamin B1 expression and increased phosphorylation of p53 and p38, accompanied by upregulation of p21 (Fig. 5G). Given the well-established role of the MAPK pathway in regulating proliferation, senescence, and apoptosis, we next investigated whether TMEM72 exerts its tumor-suppressive effects through activation of this pathway. Notably, TMEM72 overexpression significantly enhanced p38 MAPK and p53 phosphorylation, which was effectively attenuated by the p38-specific inhibitor SB202190 in RCC cells (Fig. 5H). Functionally, inhibition of p38 MAPK by SB202190 partially reversed the anti-proliferative effects of TMEM72. EdU incorporation and β-galactosidase/lysosomal staining demonstrated increased proliferation and reduced senescence in TMEM72-overexpressing RCC cells following p38 inhibition (Fig. 5I–J). To further corroborate these findings, key components of the MAPK pathway were examined in TMEM72-silenced Caki-1 cells (NC, siRNA1-TMEM72, and siRNA2-TMEM72). Western blot analysis showed no significant changes in MLK3, p-MLK3, TAK1, p-TAK1 and MKK6 expression. In contrast, MKK3, p-MKK3/p-MKK6, and p38/p-p38 levels were markedly decreased following TMEM72 knockdown (Fig. 5K).

### TMEM72 inhibited the growth of RCC cells *in vivo*

**Fig. 6:**
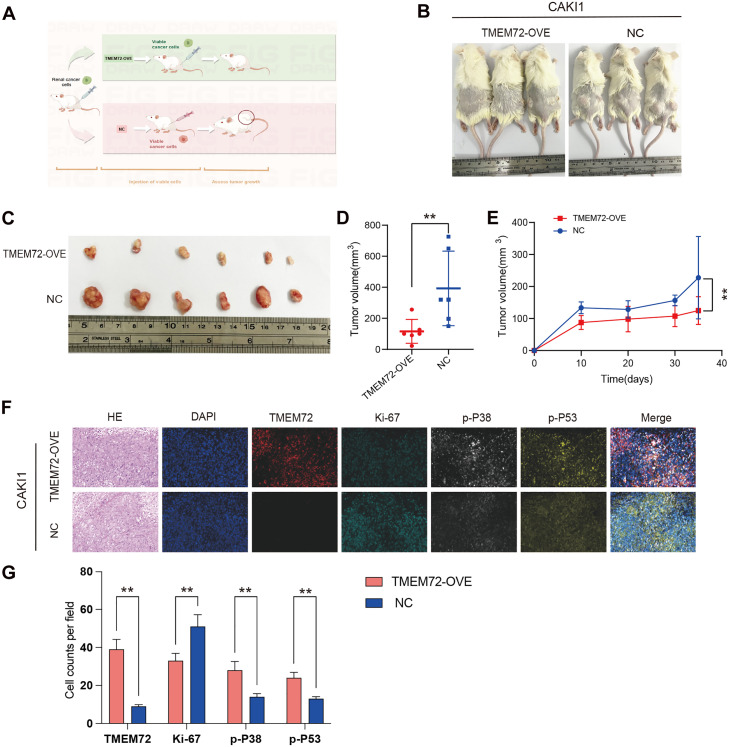
TMEM72 inhibits renal cell carcinoma growth *in vivo*. (A) Schematic illustration of the subcutaneous xenograft tumor model established in NOD/SCID mice. (B) Representative images of xenograft tumors formed by ACHN cells stably overexpressing TMEM72 or vector control following subcutaneous implantation into the dorsal flanks of NOD/SCID mice (n = 6 per group). (C) Quantification of tumor volumes in mice bearing TMEM72-overexpressing or control ACHN xenografts at the experimental endpoint. (D) Tumor growth curves showing longitudinal changes in tumor volume during the observation period in the TMEM72-overexpression and control groups. (E) Quantification of excised tumor weights from TMEM72-overexpressing and vector control groups. (F) Representative multiplex immunofluorescence images of xenograft tumor tissues showing hematoxylin and eosin (H&E) staining and immunofluorescent staining for TMEM72, Ki-67, phosphorylated p38 MAPK (p-p38), and phosphorylated p53 (p-p53), with DAPI used for nuclear counterstaining. (G) Quantification of immunofluorescence signals for TMEM72, Ki-67, p-p38, and p-p53 from the images shown in panel (F).

**Fig. 7:**
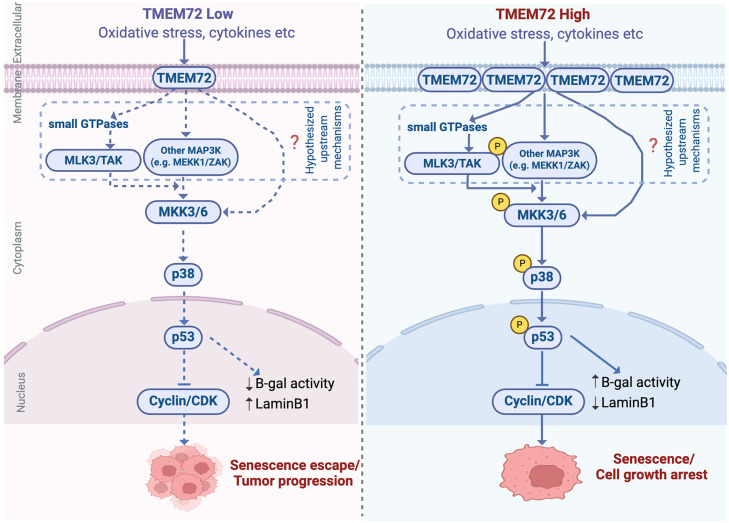
Schematic illustration of the tumor-suppressive mechanism mediated by TMEM72 in renal cell carcinoma (RCC). TMEM72 regulates renal cancer cell fate through the p38/MAPK–p53 signaling pathway: low TMEM72 expression results in insufficient pathway activation, promoting senescence escape and tumor progression, whereas high TMEM72 expression activates the signaling cascade, inducing cellular senescence and growth arrest.

A subcutaneous xenograft model was generated to evaluate TMEM72’s role in RCC progression *in vivo*. Caki-1 cells stably overexpressing TMEM72 or vector controls were injected into the bilateral dorsal flanks of NOD/SCID mice (Fig. 6A). Representative images of tumor-bearing mice and excised tumors revealed substantially smaller tumors in the TMEM72-overexpressing group compared with controls (Fig. 6B-5C). Quantitative analysis further confirmed that TMEM72 overexpression significantly decreased tumor volume *in vivo* (Fig. 6D). Consistently, longitudinal monitoring revealed that tumors derived from TMEM72-overexpressing cells grew at a significantly slower rate than control tumors, as reflected by the tumor growth curves (Fig. 6E). In line with the *in vitro* results, Ki-67 levels were substantially lower in TMEM72-overexpressing tumors than in controls, accompanied by elevated phosphorylation of p53 and p38 MAPK, suggesting *in vivo* activation of the p38 MAPK-p53 pathway (Fig. 6F-J). Collectively, these findings support a model in which TMEM72 exerts tumor-suppressive effects in RCC (Fig. 7). Elevated TMEM72 activates the MAPK signaling pathway, promoting the phosphorylation and nuclear activation of p53. This, in turn, induces cellular senescence, leading to G1/S cell-cycle arrest and ultimately suppressing RCC cell proliferation.

## Discussion

RCC is among the most frequently diagnosed cancers globally and is characterized by a subtle onset and highly aggressive biology. Despite advances in diagnostic and therapeutic strategies, the molecular mechanisms underlying RCC progression remain incompletely understood, which is reflected by the limited availability of effective therapeutic targets and reliable prognostic biomarkers [26]. Therefore, the identification of novel molecular markers that can improve risk stratification and therapeutic intervention in RCC is urgently needed [27]. Accumulating evidence suggests that transmembrane proteins play critical roles in cancer initiation and progression and may serve as promising prognostic biomarkers or therapeutic targets in various malignancies [5]. Although TMEM72, a transmembrane protein family member, is known to play important roles in renal biology, its function and clinical relevance in RCC have not been characterized.

Recent studies have emphasized the importance of identifying robust prognostic biomarkers in RCC. For example, a pan-cancer analysis demonstrated that dysregulated genes such as leucine zipper protein 2 are closely associated with tumor progression and patient survival [28]. Consistent with these findings, our analysis revealed that TMEM72 is commonly downregulated in RCC tissues, and this decreased expression correlates with more advanced pathological stage and worse patient prognosis. Notably, high TMEM72 expression was correlated with prolonged survival, indicating that TMEM72 may serve as an independent prognostic factor for RCC patients. While TMEM72 has been previously associated with RCC, how its dysregulation contributes to disease progression has not been elucidated [29]. Functionally, we found that loss of TMEM72 promoted tumorigenesis and enhanced proliferative capacity in RCC cells. Given the critical role of cell cycle dysregulation in RCC, transcriptomic profiling revealed that TMEM72 overexpression is significantly associated with pathways involved in cell cycle control, senescence, and MAPK signaling [[30], [31], [32]]. These results suggest that TMEM72 may suppress tumor growth by modulating cell cycle–related processes, although the exact underlying mechanisms remain to be elucidated. Senescence constitutes a stable and irreversible cessation of cell proliferation and acts as an important barrier against tumorigenesis in multiple cancer types [33,34]. Several transmembrane proteins, such as BNIP3 and PLA2R1, have been reported to positively regulate cellular senescence [35,36]. Based on these observations, we hypothesized that TMEM72 might also participate in the regulation of senescence in RCC. Consistent with this hypothesis, RNA-seq based heatmap and enrichment analyses revealed significant enrichment of senescence-associated gene signatures in TMEM72-overexpressing cells. Experimental validation further confirmed that TMEM72 overexpression suppressed cell cycle-related markers while markedly increasing senescence-associated β-galactosidase activity. These results indicate that TMEM72 inhibits RCC cell proliferation, at least in part, by promoting cellular senescence.

Increasing evidence indicates that the tumor immune microenvironment plays a pivotal role in RCC progression, and immune-related signatures have shown strong prognostic value [37]. In line with these observations, our findings reveal that TMEM72 expression is closely associated with distinct immune infiltration patterns, suggesting its potential involvement in shaping the immune microenvironment. Based on our analysis results of immune infiltration, the high expression of TMEM72 is significantly correlated with the enrichment of anti-tumor immune cells such as M1-type macrophages, monocytes, and NK cells, while it is negatively correlated with immunosuppressive cells such as Tregs and M0-type macrophages. Increasing evidence indicates that the tumor immune microenvironment plays a pivotal role in RCC progression, and immune-related signatures have shown strong prognostic value [38]. In line with these observations, our findings reveal that TMEM72 expression is closely associated with distinct immune infiltration patterns, suggesting its potential involvement in shaping the immune microenvironment.

Importantly, our study uncovered a previously unrecognized mechanism by which TMEM72 activates the tumor-suppressive p38 MAPK signaling pathway, leading to the induction of cellular senescence in RCC. MAPK signaling orchestrates multiple critical cellular functions, such as cell cycle progression, apoptosis, senescence, and cancer development [39,40]. The tumor-suppressive effects of p38 MAPK are primarily mediated by the p38α and p38β isoforms, which inhibit cell cycle checkpoints at G0, G1/S, and G2/M phases, leading to growth arrest, apoptosis, or senescence [41]. Previous studies have shown that p38 MAPK modulates p53 activity through post-translational modifications, resulting in p53 stabilization and activation [42]. ince cellular senescence is a key mediator of p53-dependent tumor suppression, activation of the p38–p53 axis constitutes an important antitumor mechanism [43].

Indeed, p38 MAPK has been reported to regulate p53 either directly or indirectly by preventing Mdm2-mediated degradation, leading to p53 accumulation and subsequent induction of the G1/S checkpoint [44,45]. However, the upstream regulatory signals that activate the p38 MAPK pathway remain largely undefined. Emerging evidence indicates that alterations in transmembrane proteins can modulate MAPK signaling [46]. In line with this notion, our data demonstrate that TMEM72 overexpression markedly enhances p38 phosphorylation and downstream p53 activation, whereas TMEM72 silencing or pharmacological inhibition of p38 attenuates these effects. These results indicate that TMEM72 acts as an upstream regulator of the p38 MAPK pathway, promoting cellular senescence and inhibiting RCC progression.

Nevertheless, the precise molecular mechanisms by which TMEM72 activates p38 MAPK signaling remain to be elucidated. Further investigations are needed to identify the upstream signaling events and interacting partners that connect TMEM72 to MAPK activation. Understanding these mechanisms could provide new insights into RCC pathogenesis and support TMEM72 as a potential therapeutic target.

## Conclusion

Our findings identify TMEM72 as a tumor suppressor in RCC, negatively associated with tumor proliferation and patient prognosis. Mechanistically, its antitumor effects are mediated at least in part through MAPK pathway activation, highlighting TMEM72 as a potential prognostic biomarker and therapeutic target.

## Data availability

The datasets generated and analyzed during this study are available from the corresponding author upon reasonable request.

## Ethical statement

Human RCC tissues were obtained from patients undergoing surgical resection at Peking University Shenzhen Hospital (Shenzhen, China) with approval from the Institutional Ethics Committee (No. 2023-184-1). All animal experiments were conducted in accordance with guidelines approved by the Animal Ethics Committee of Shenzhen PKU-HKUST Medical Center (No. 2023-425).

## Funding

This work was supported by grants from the following funding agencies: Basic and Applied Basic Research Funding of Guangdong Province (2023A1515220164), Shenzhen Medical Research Fund (A2503095), Natural Science Foundation of Shenzhen Science and Technology Innovation Committee (JCYJ20220531093800001), National Natural Science Foundation of China (82472831, 82073182), Natural Science Foundation of Guangdong Province (2024A1515010330), Shenzhen Clinical Research Center for Urology and Nephrology (LCYSSQ20220823091403008).

## Declaration of generative AI and AI-Assisted technologies in the manuscript preparation process

During the preparation of this work, the author(s) used DeepSeek and Grammarly for language editing and improvement. After using these tools, the author(s) carefully reviewed and revised the content as necessary and take full responsibility for the integrity and accuracy of the published article.

## CRediT authorship contribution statement

**Fang Dai:** Writing – review & editing, Writing – original draft, Software, Methodology, Investigation, Formal analysis, Data curation. **Huming Wang:** Writing – review & editing, Validation, Supervision, Software, Formal analysis, Data curation. **Wenhao Chu:** Writing – review & editing, Software, Methodology, Data curation, Investigation. **Yu Lin:** Writing – review & editing, Writing – original draft, Software, Investigation, Formal analysis. **Jinqing He:** Writing – review & editing, Validation, Resources, Methodology. **Hantao Wen:** Writing – review & editing, Software, Funding acquisition. **Xiaotong Feng:** Writing – review & editing, Validation, Software. **Xudong Liu:** Writing – review & editing, Funding acquisition, Formal analysis. **Zihan Xu:** Writing – review & editing, Software, Methodology, Investigation. **Liangkuan Bi:** Writing – review & editing, Validation, Supervision, Project administration, Methodology, Funding acquisition. **Zhaojie Lyu:** Writing – review & editing, Writing – original draft, Project administration, Funding acquisition, Formal analysis, Data curation, Conceptualization.

## Declaration of competing interest

The authors declare that they have no known competing financial interests or personal relationships that could have appeared to influence the work reported in this paper.

## Appendix Supplementary materials

Image, application 1
