# Supplementary material for: TMEM72 Inhibits the proliferation by promoting cellular senescence through the activation of the P38/MAPK signaling pathway in renal cell carcinoma
Source: Transl Oncol. 2026 May 2;69:102789. doi: 10.1016/j.tranon.2026.102789 (PMC13147424; doi:10.1016/j.tranon.2026.102789)
Supplement: Supplementary file 1 [file mmc1.pdf]

Supplementary Materials
Fig 1H

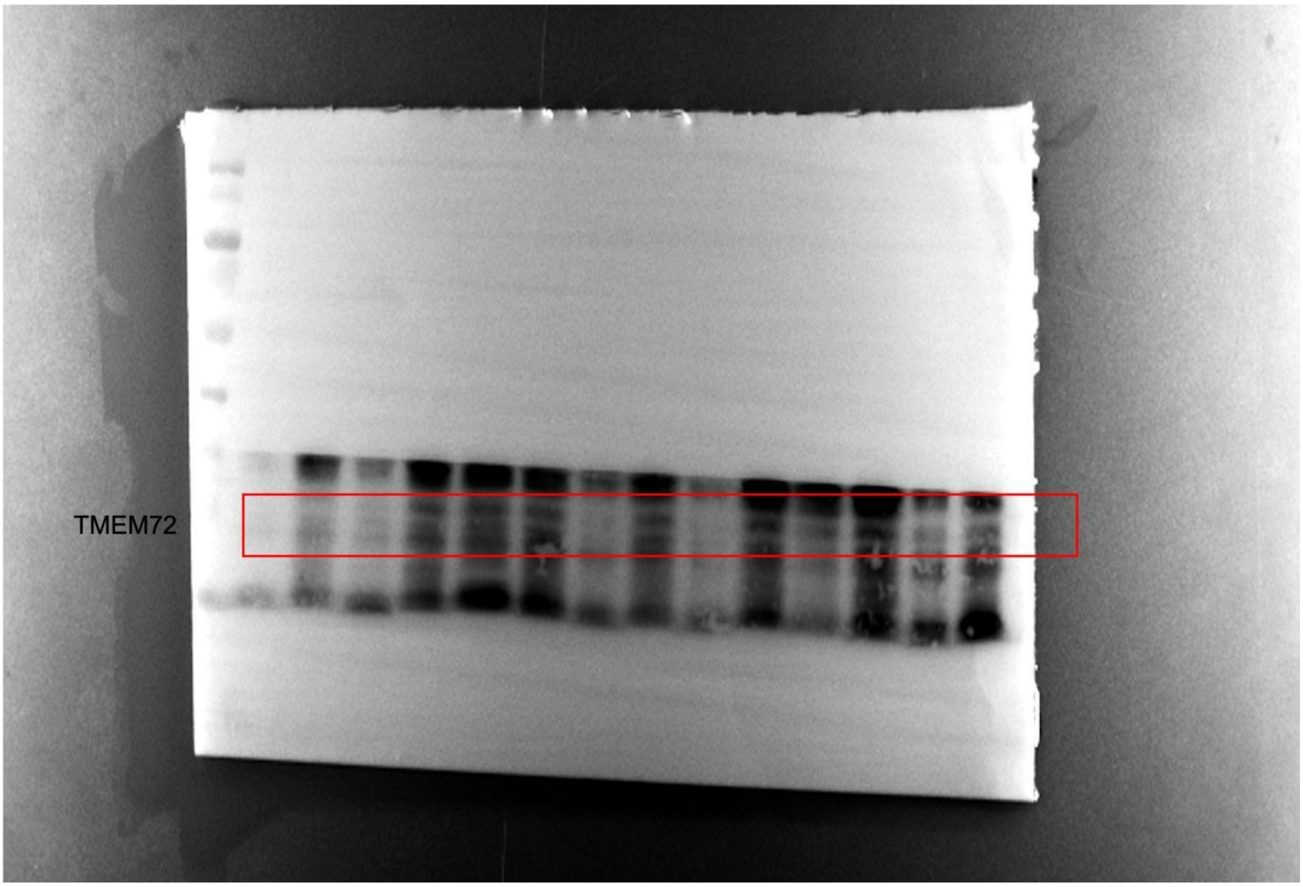

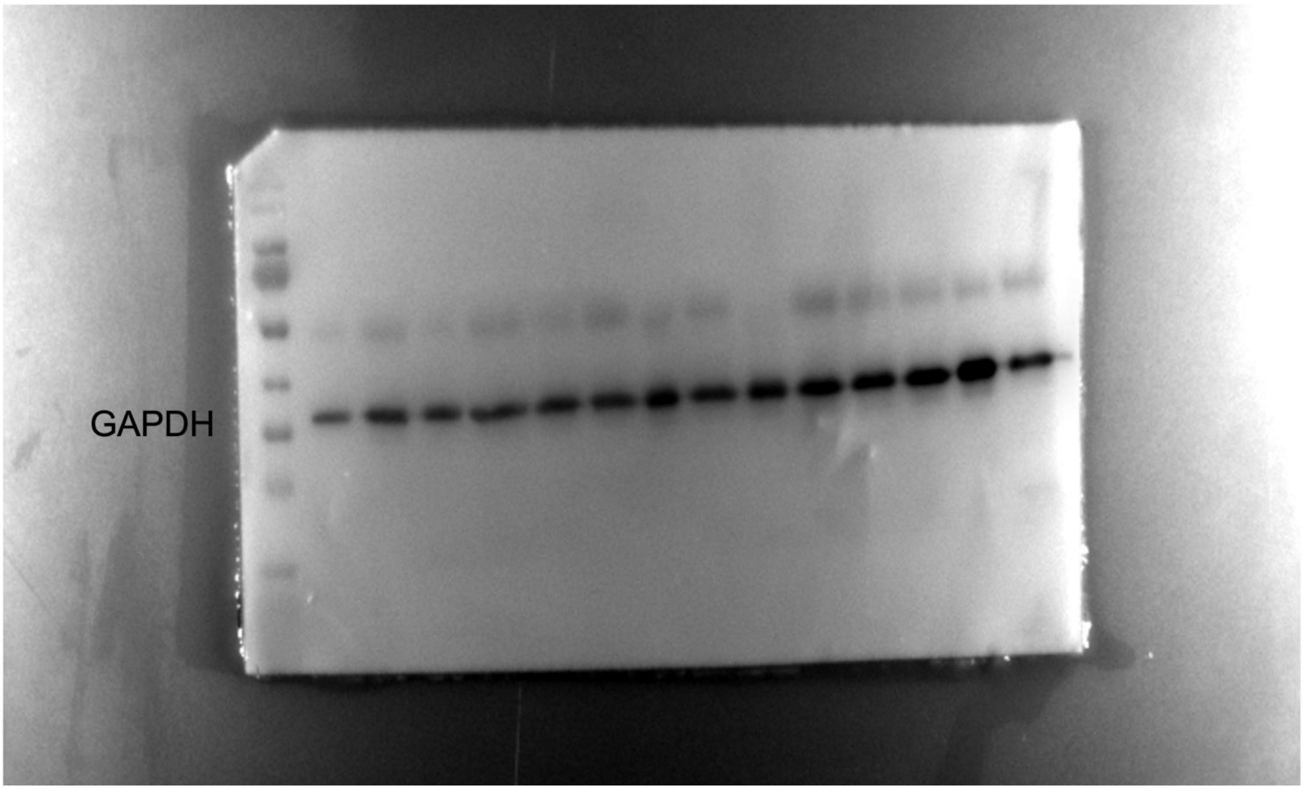

Fig 2B

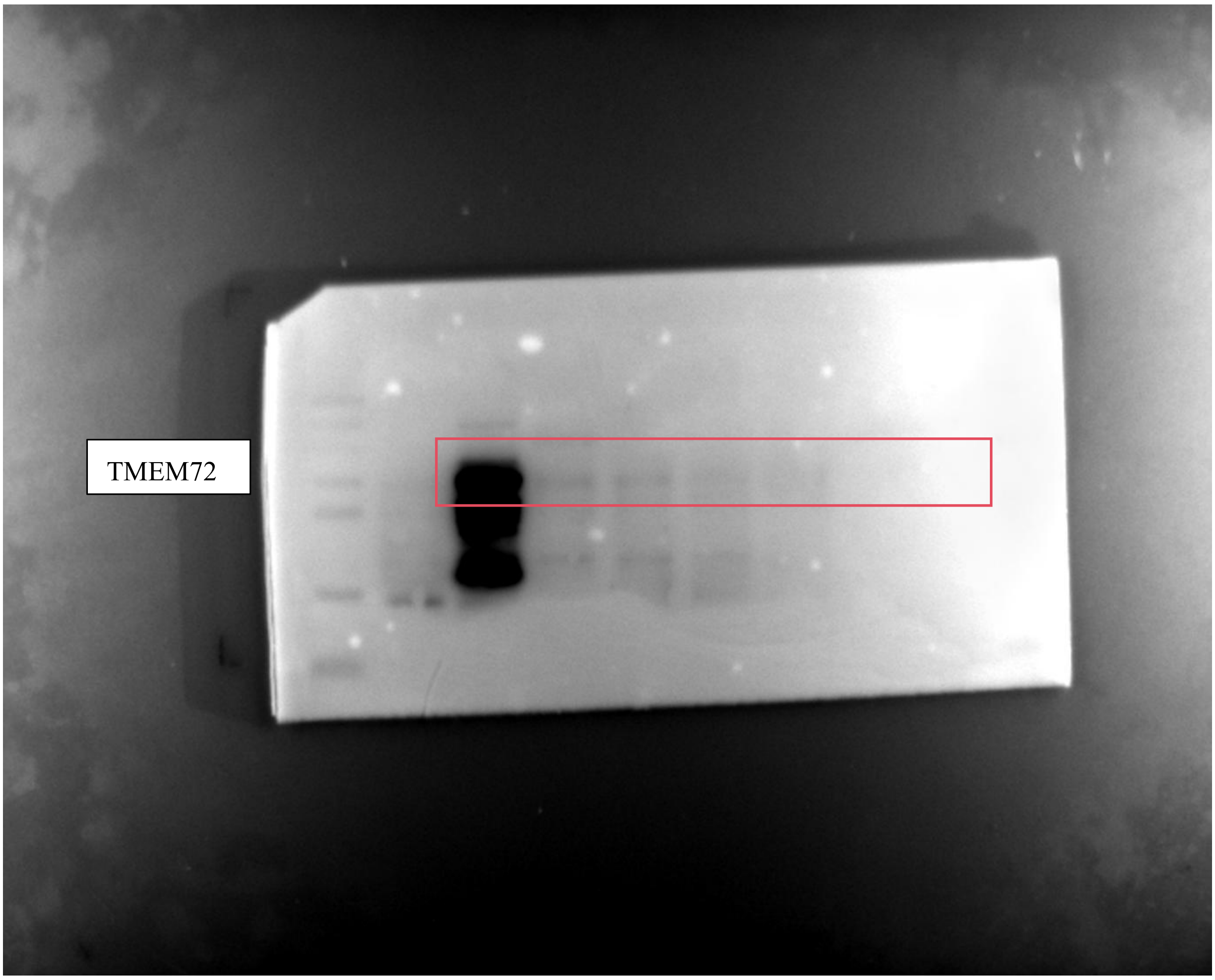

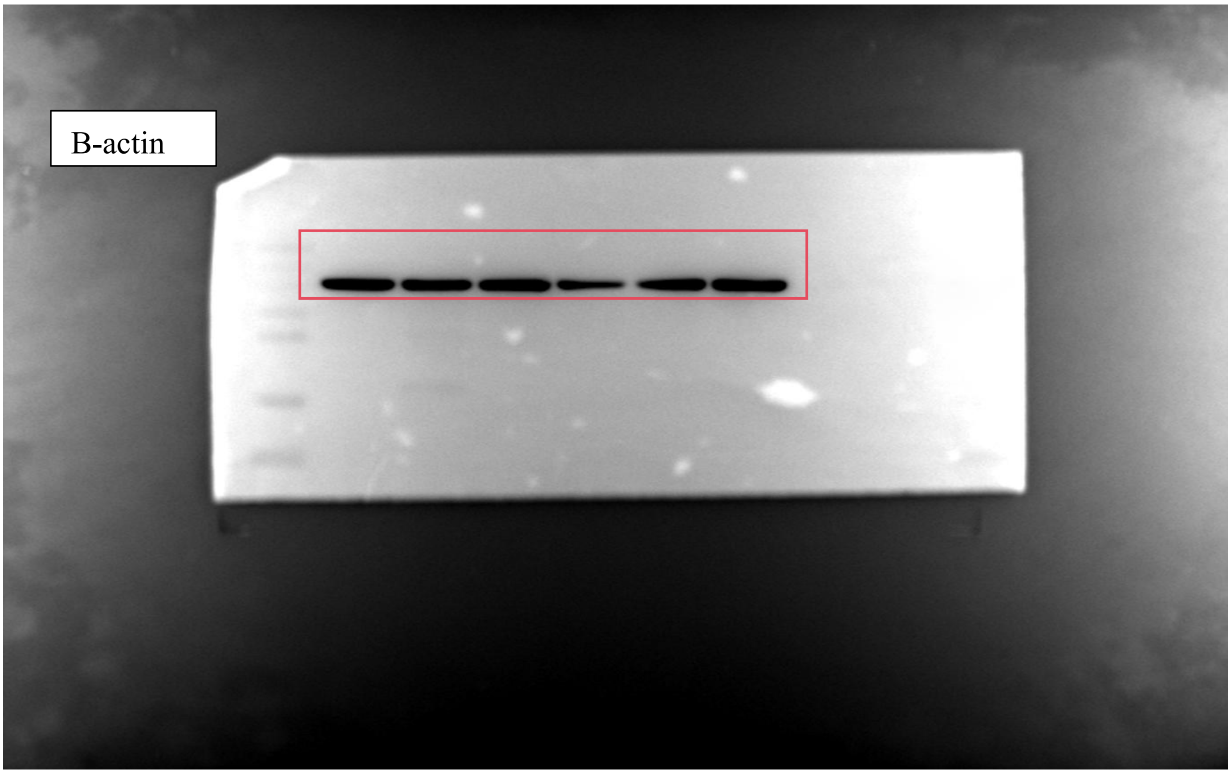

Fig 3D

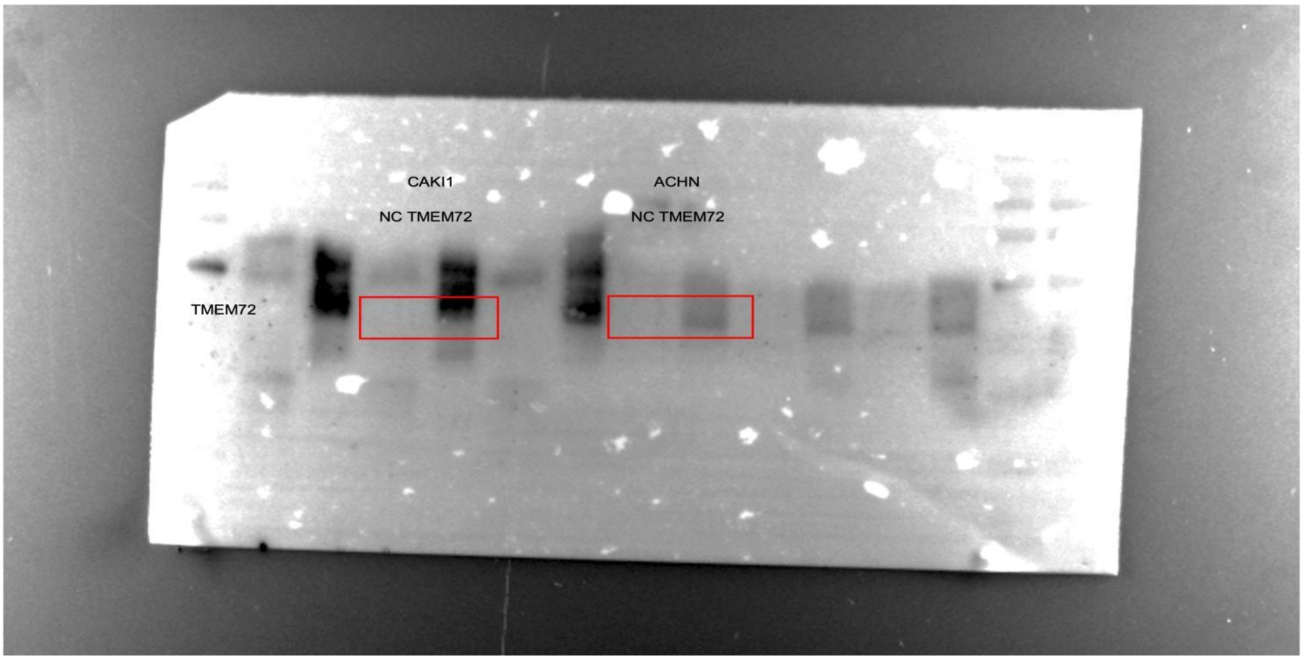
CAKI1
NC TMEM72
ACHN
NC TMEM72
TMEM72

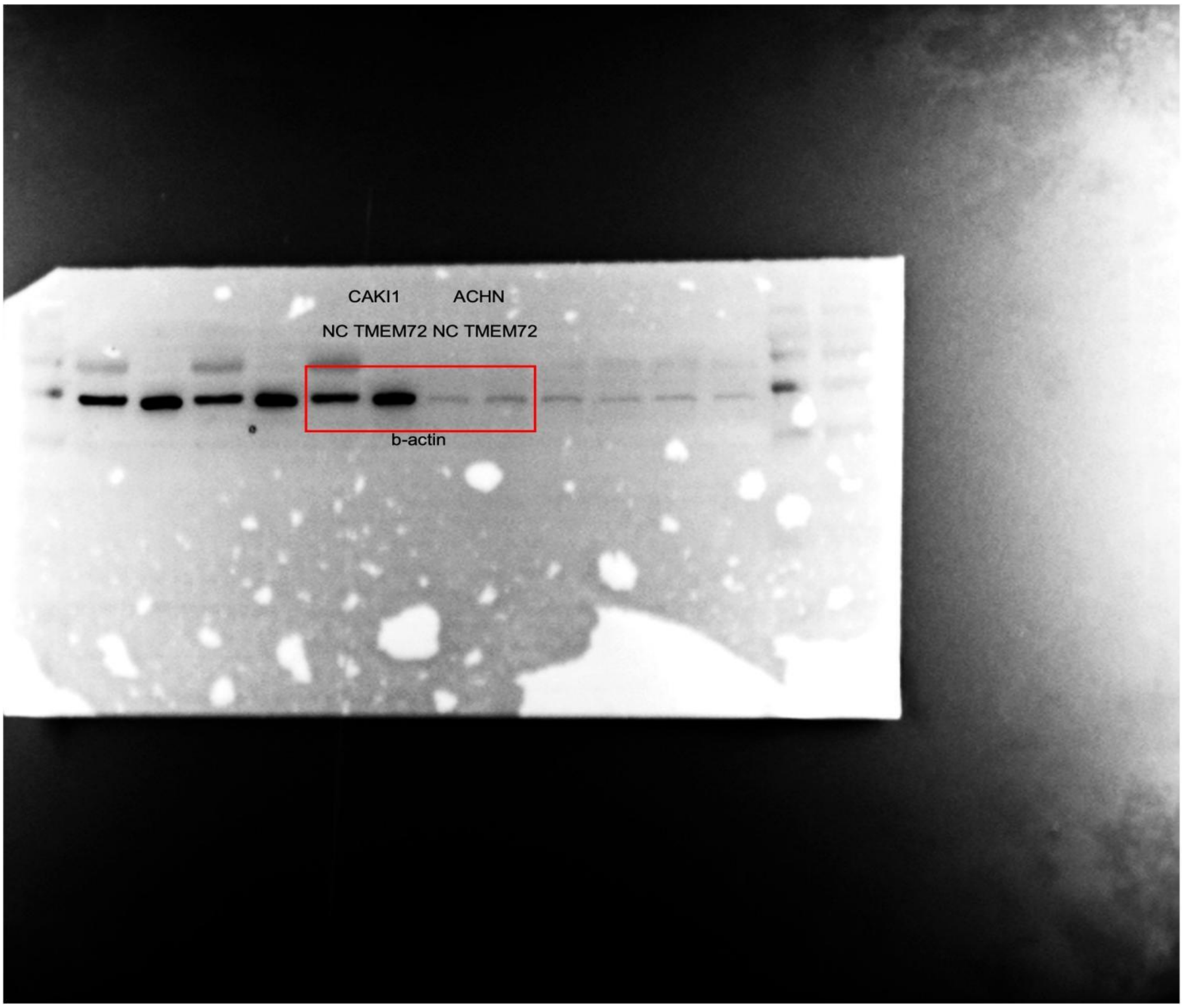
CAKI1
ACHN
NC TMEM72 NC TMEM72
b-actin

Fig 4B

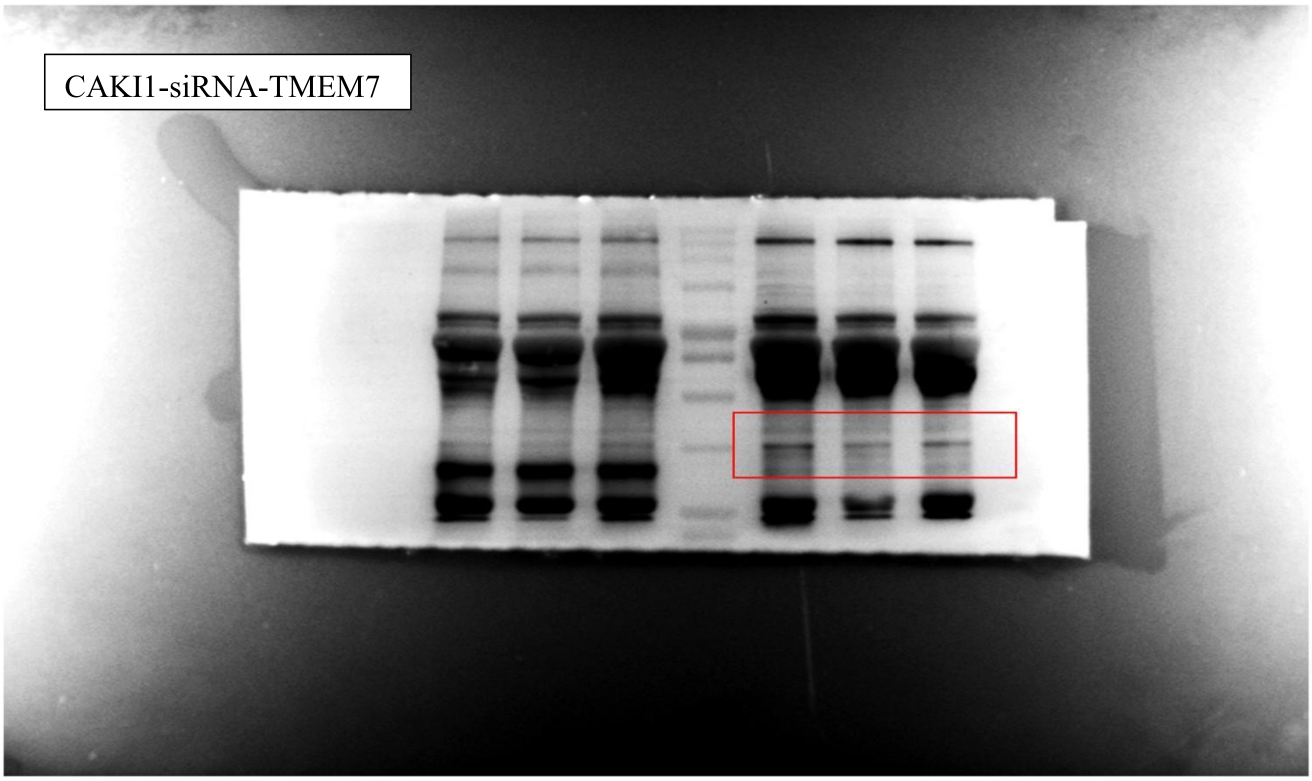

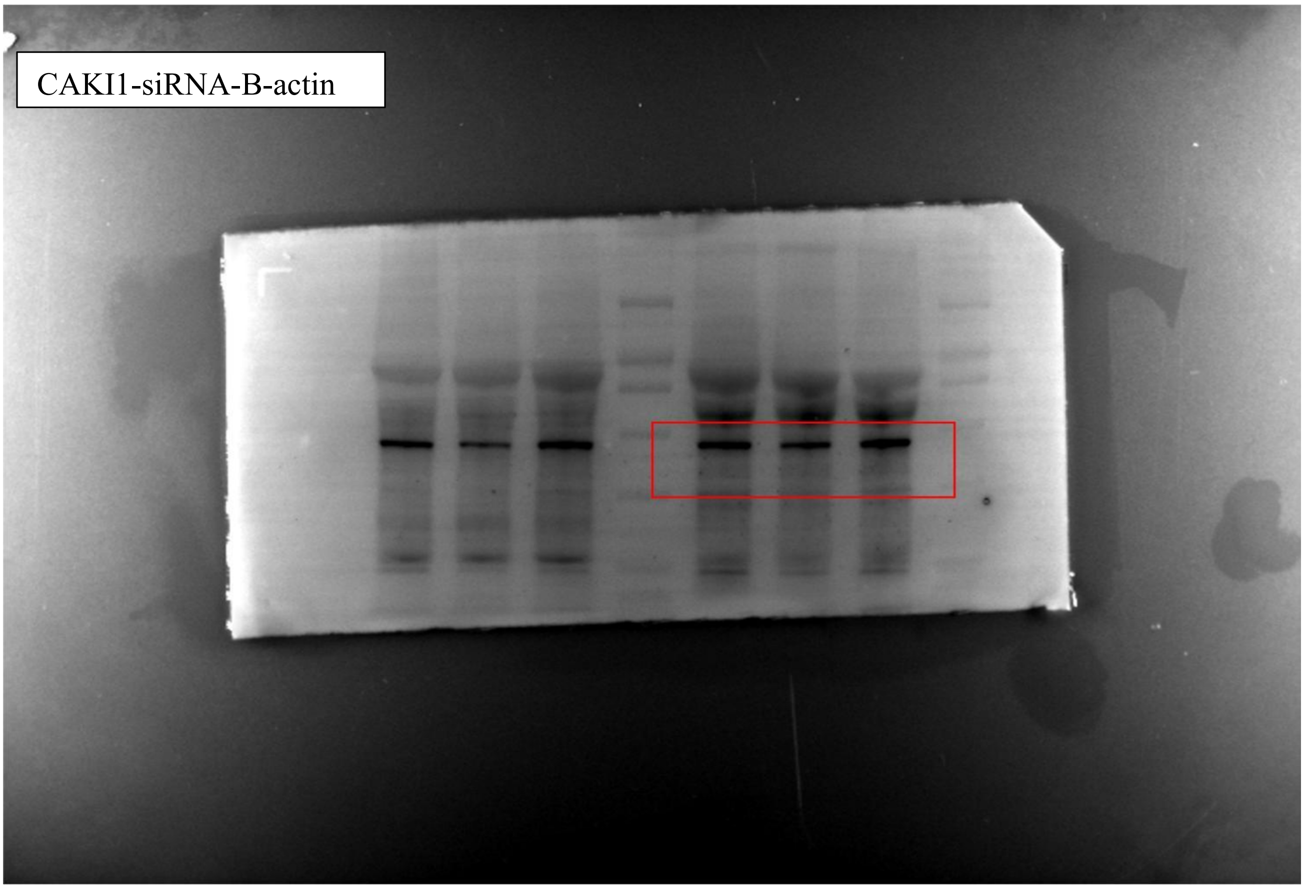

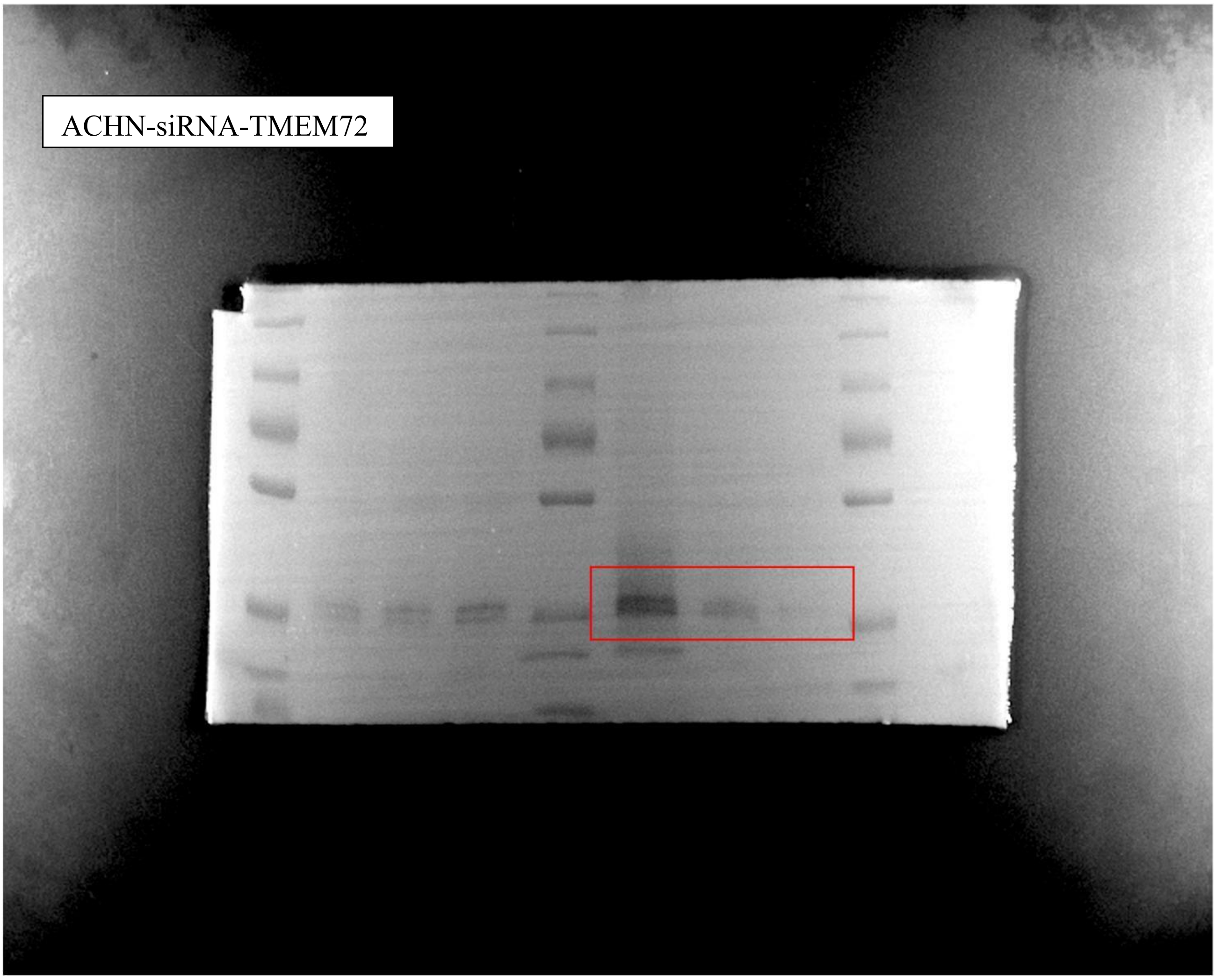
ACHN-siRNA-TMEM72

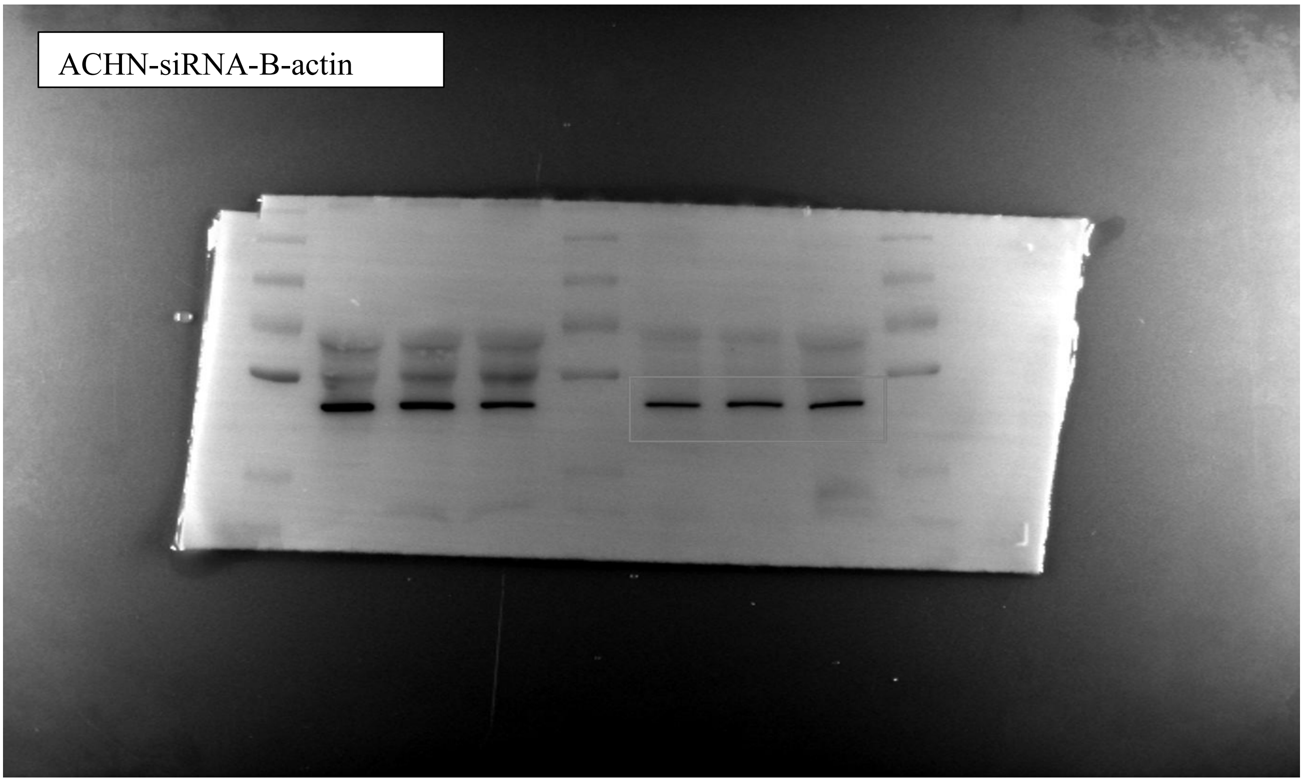
ACHN-siRNA-B-actin

Fig 5G

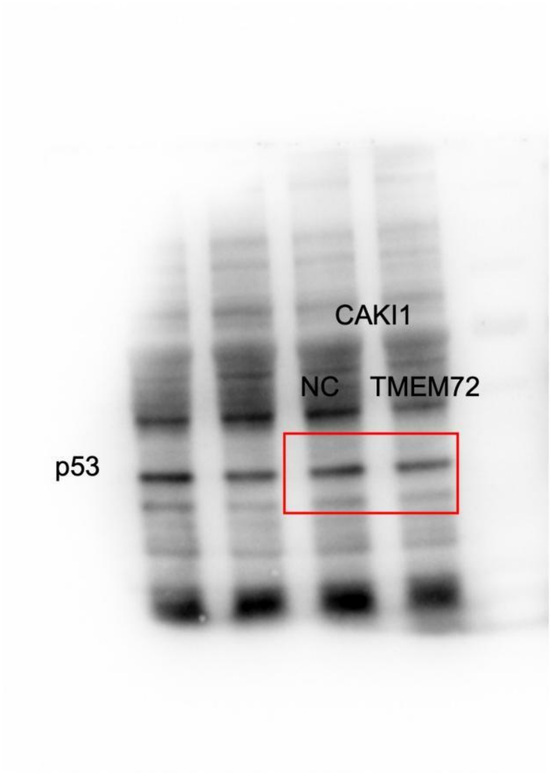

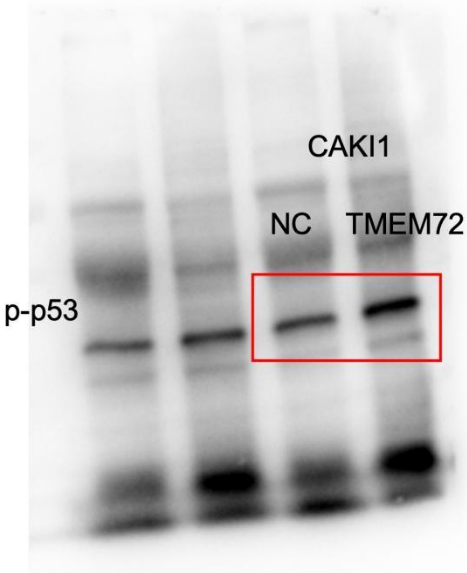

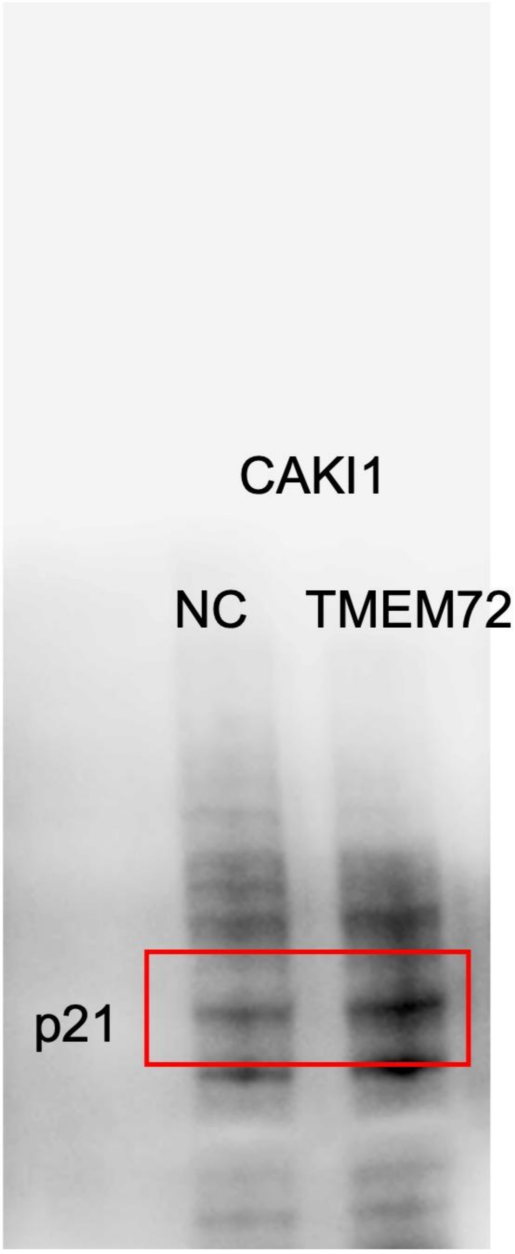
CAKI1
NC
TMEM72
p21

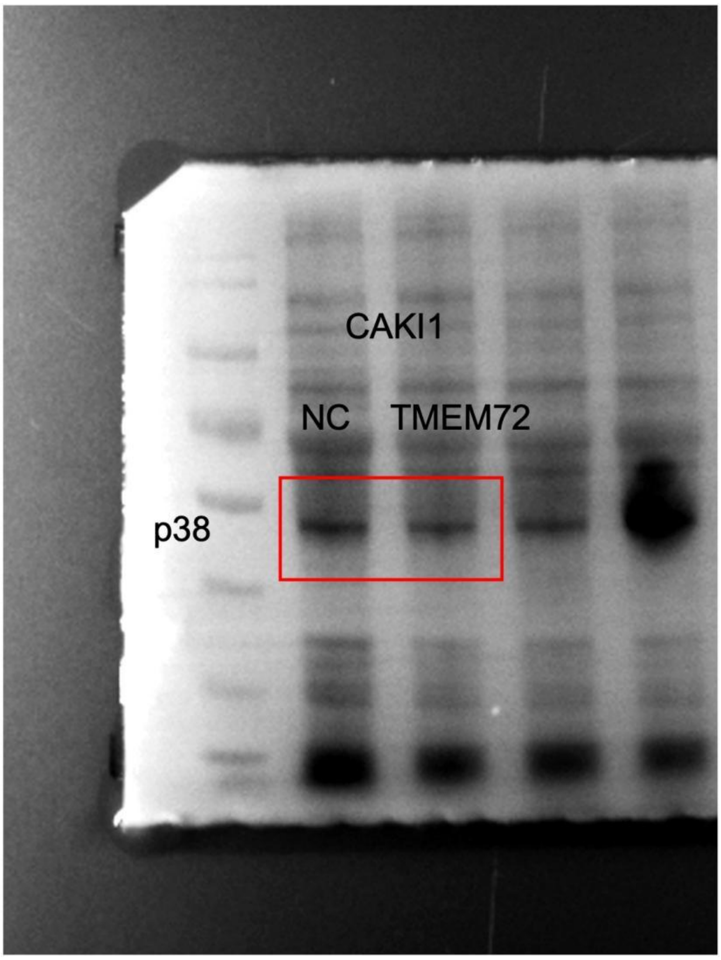
CAKI1
NC
TMEM72
p38

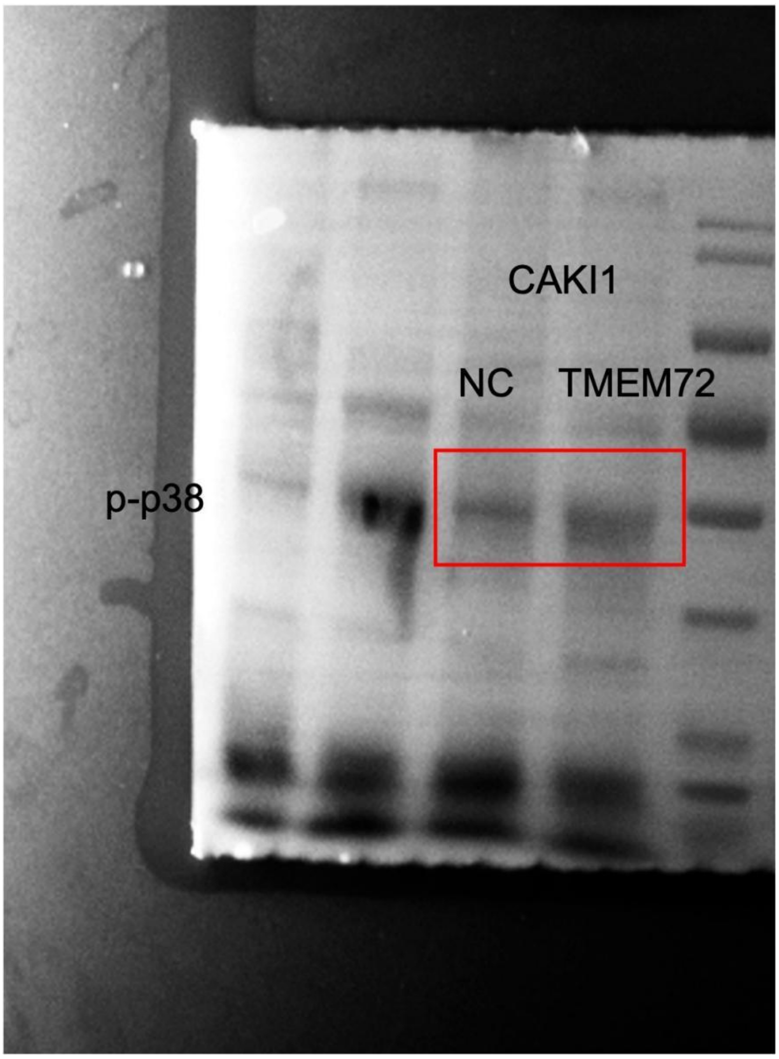
CAKI1
NC
TMEM72
p-p38

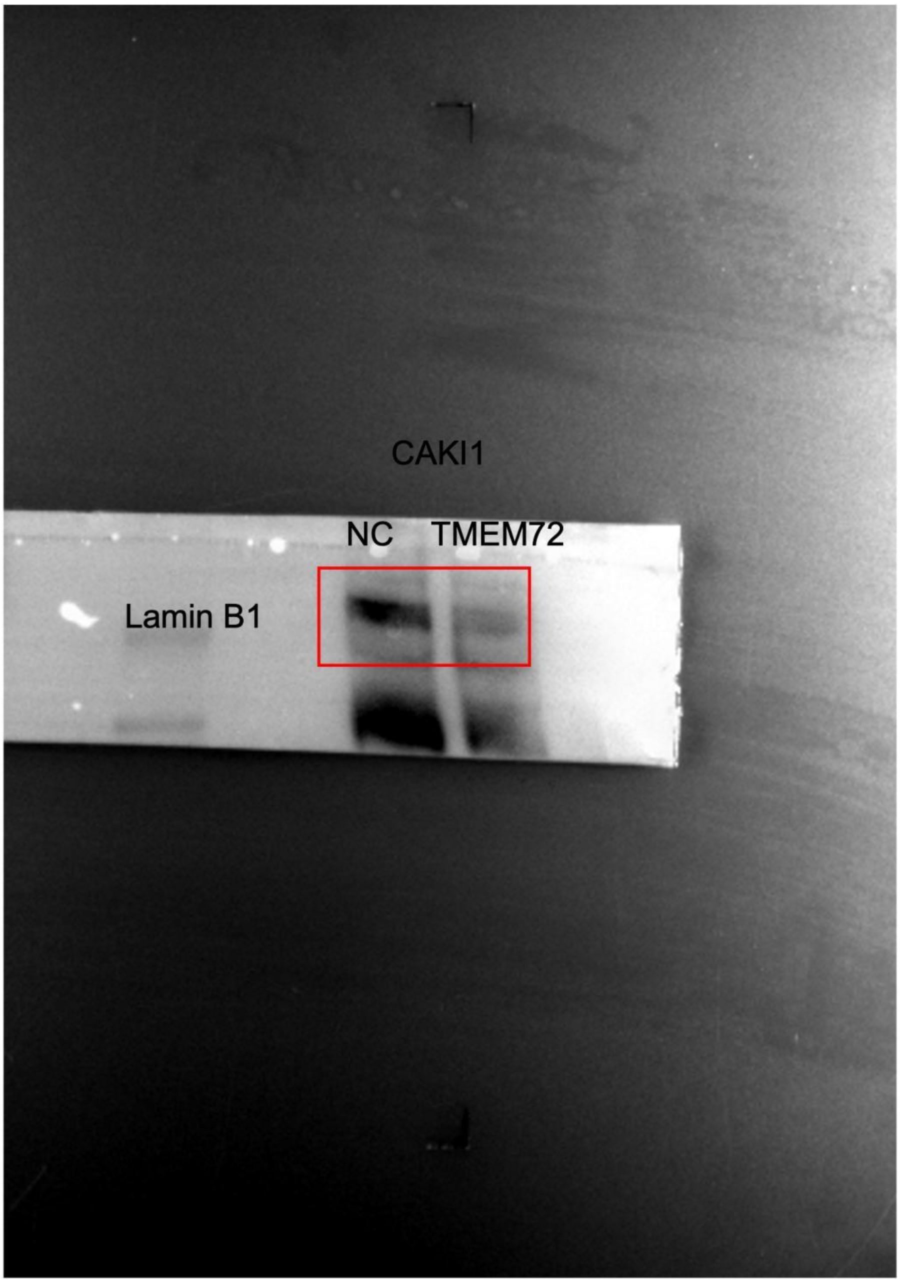
CAKI1
NC
TMEM72
Lamin B1

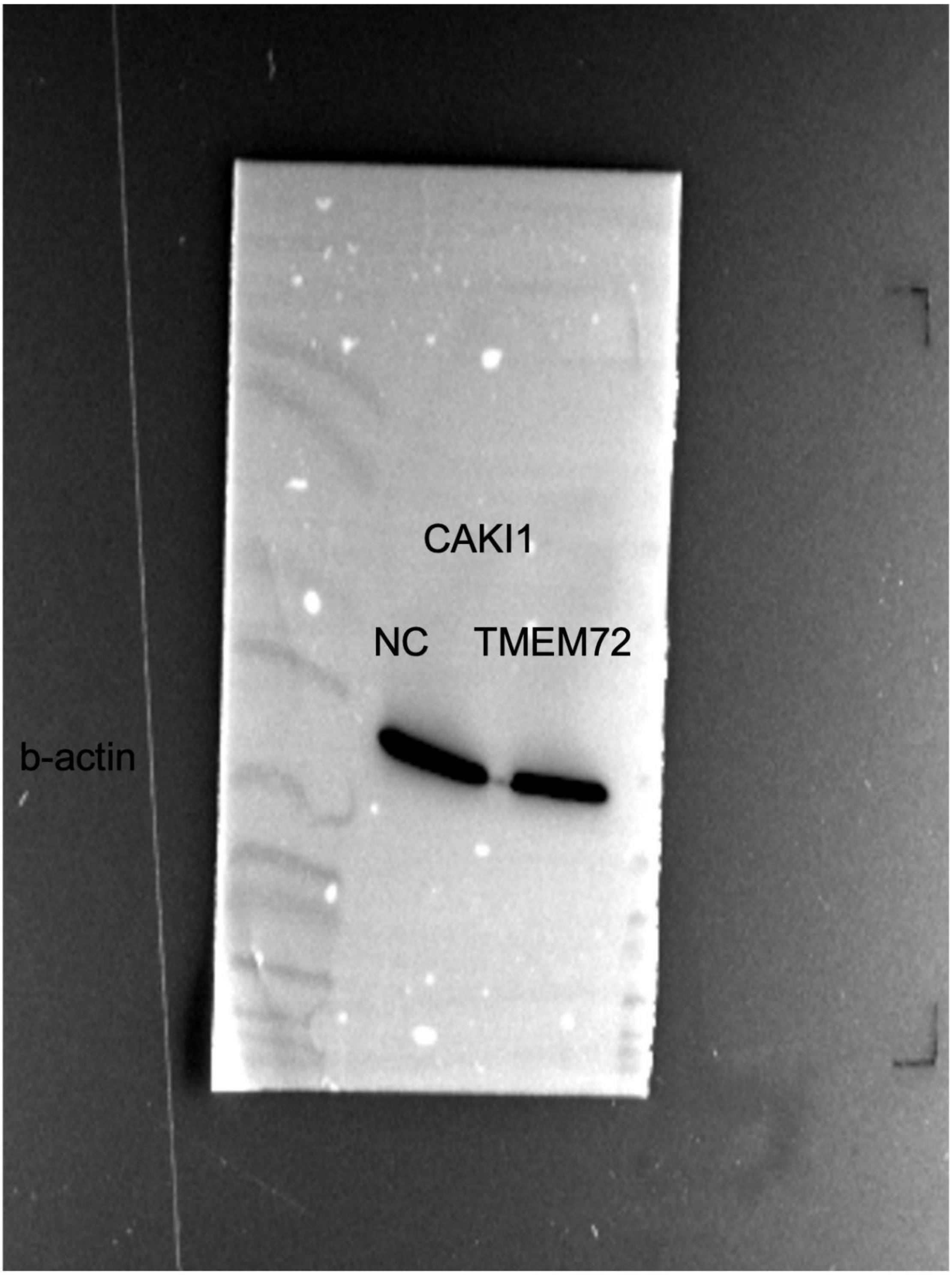
CAKI1
NC
TMEM72
b-actin

Fig 5H

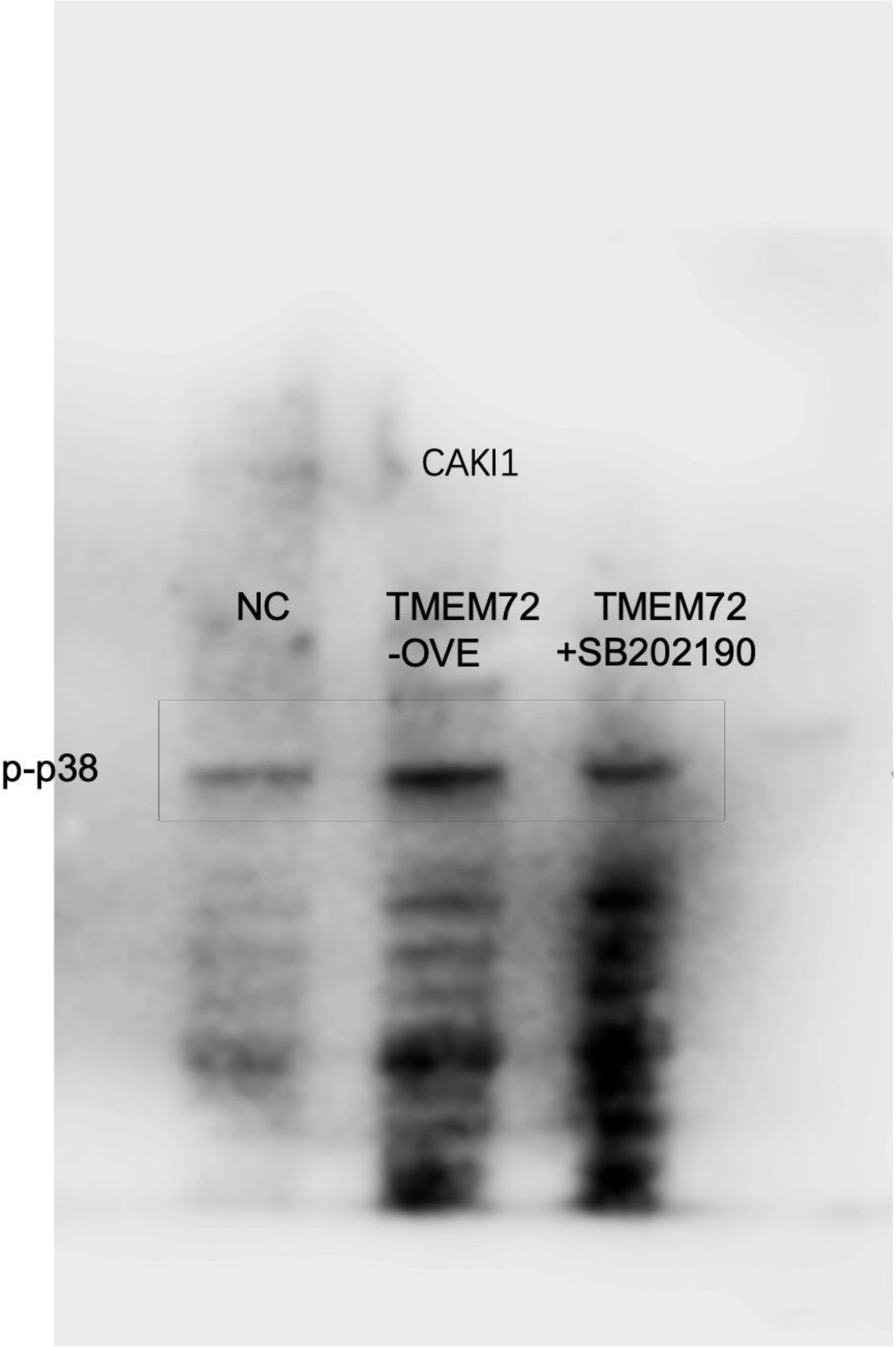

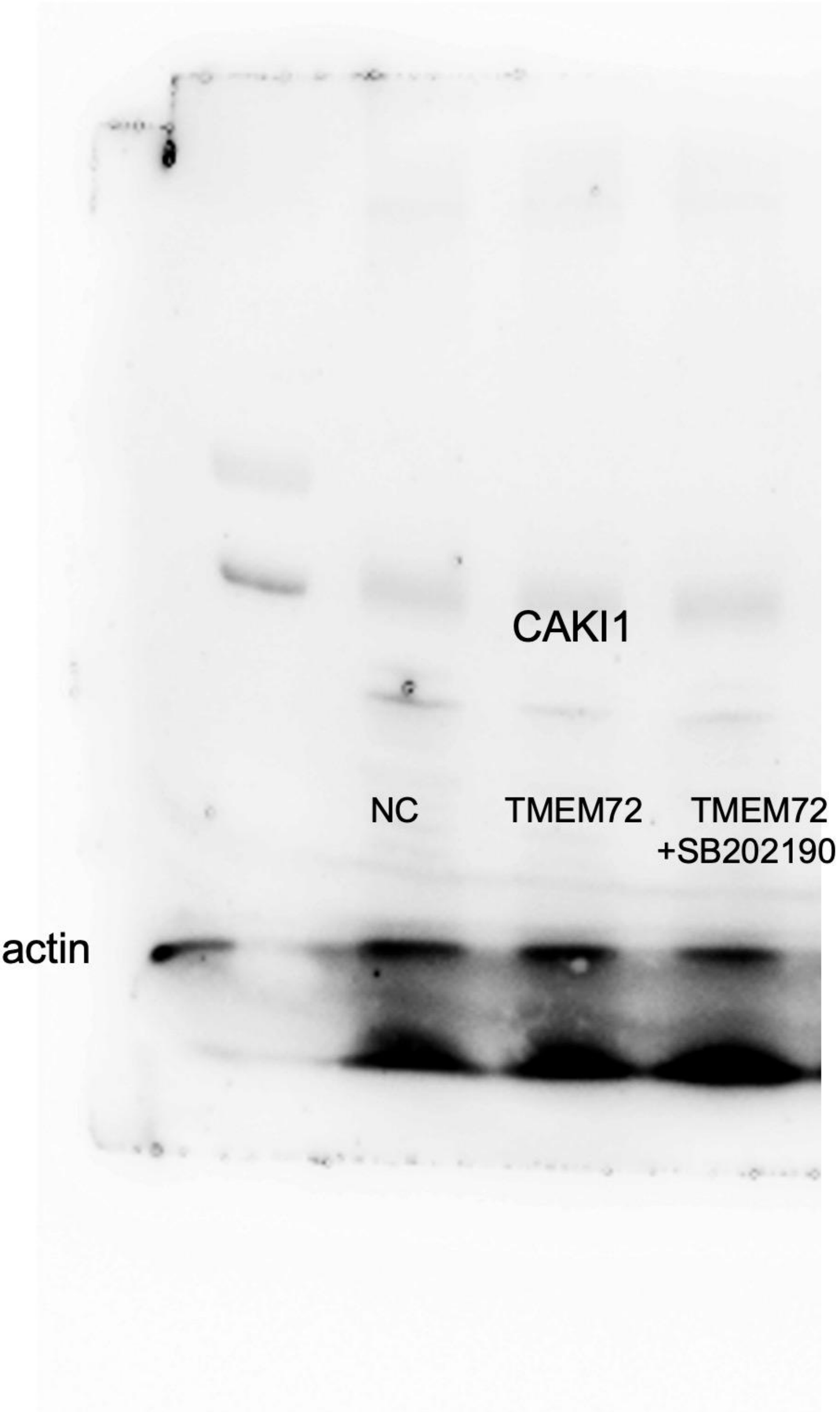
CAKI1
NC
TMEM72
TMEM72
+SB202190
actin

Fig 5K

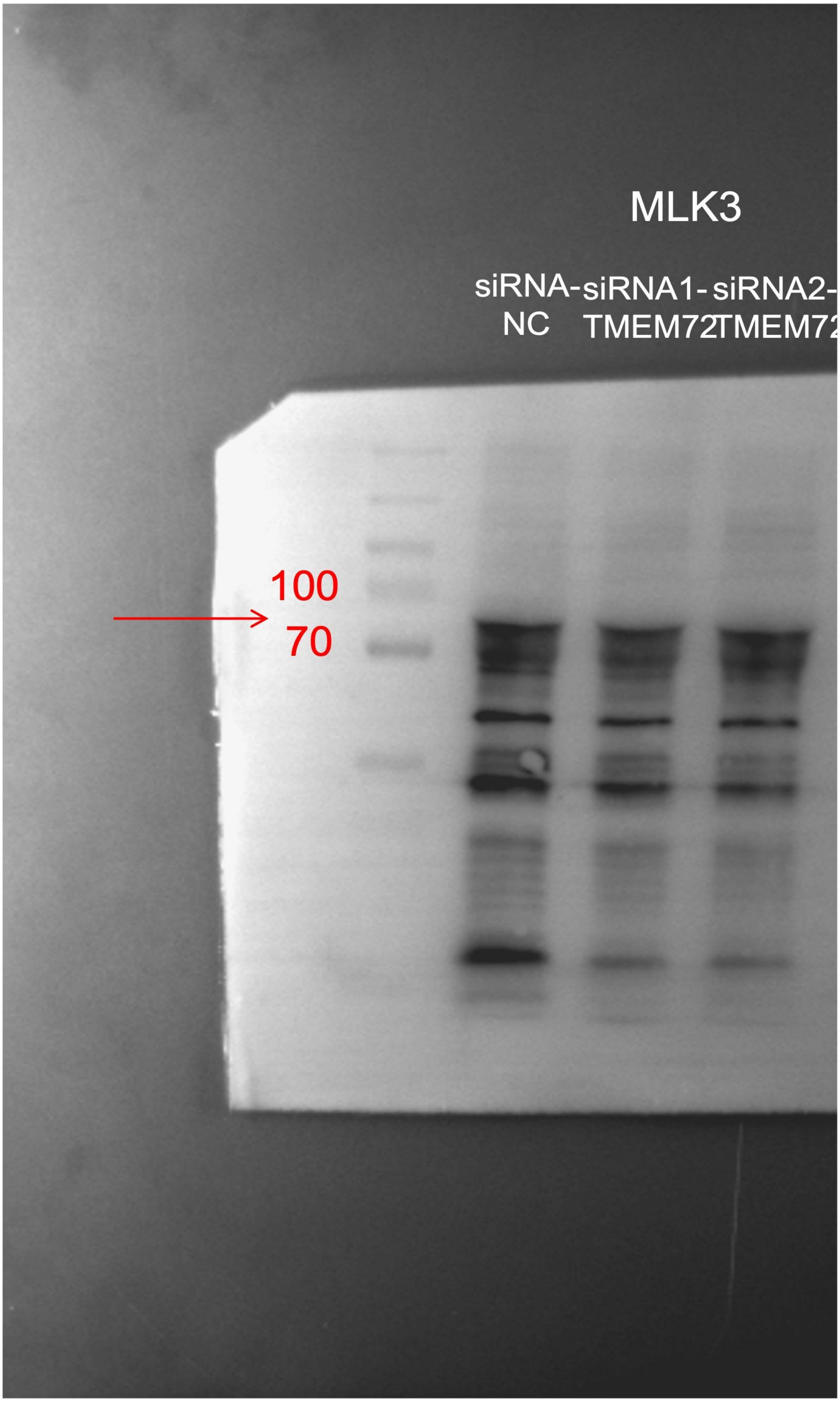

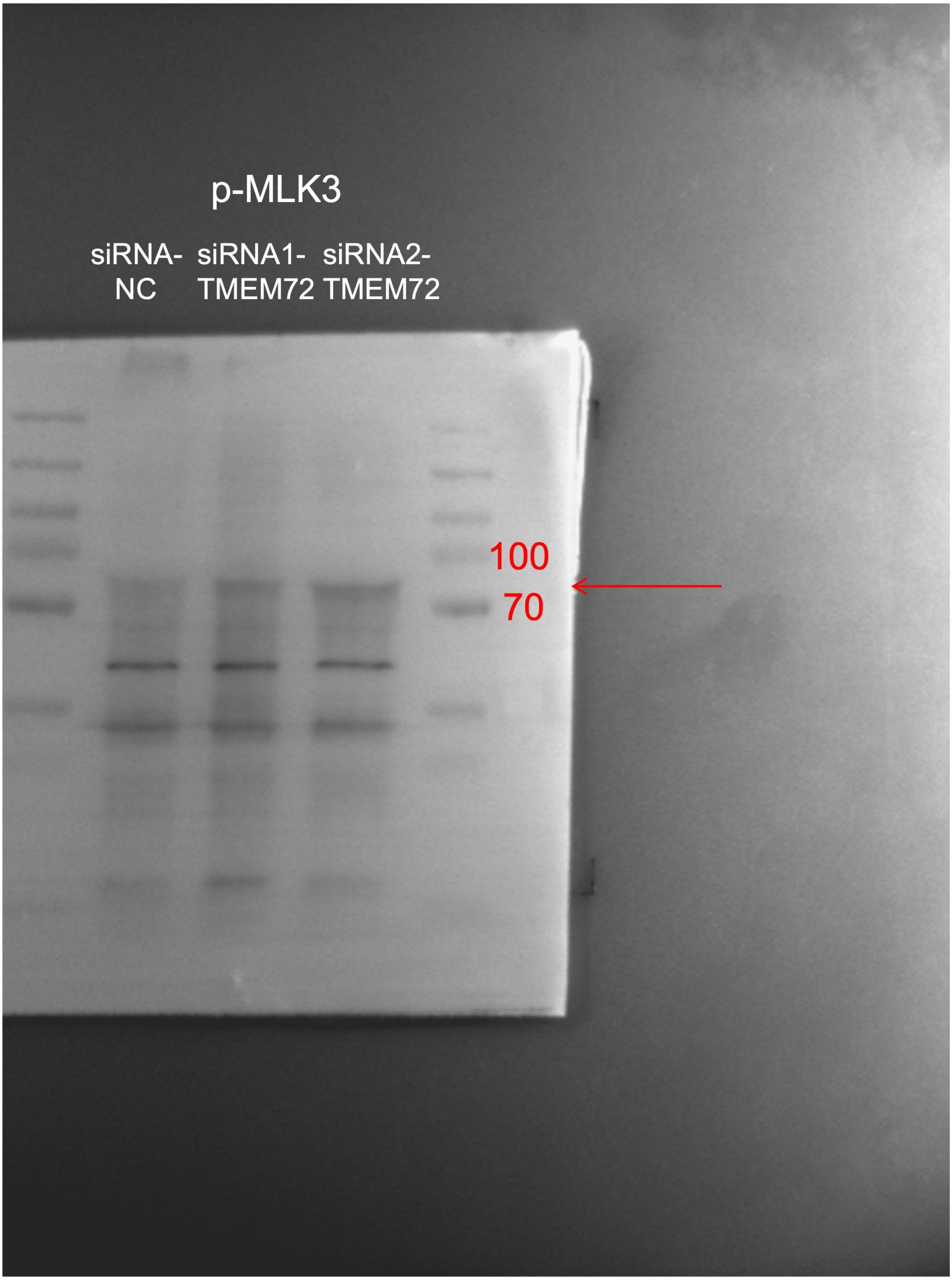
p-MLK3
siRNA-NC
siRNA1-TMEM72
siRNA2-TMEM72
100
70

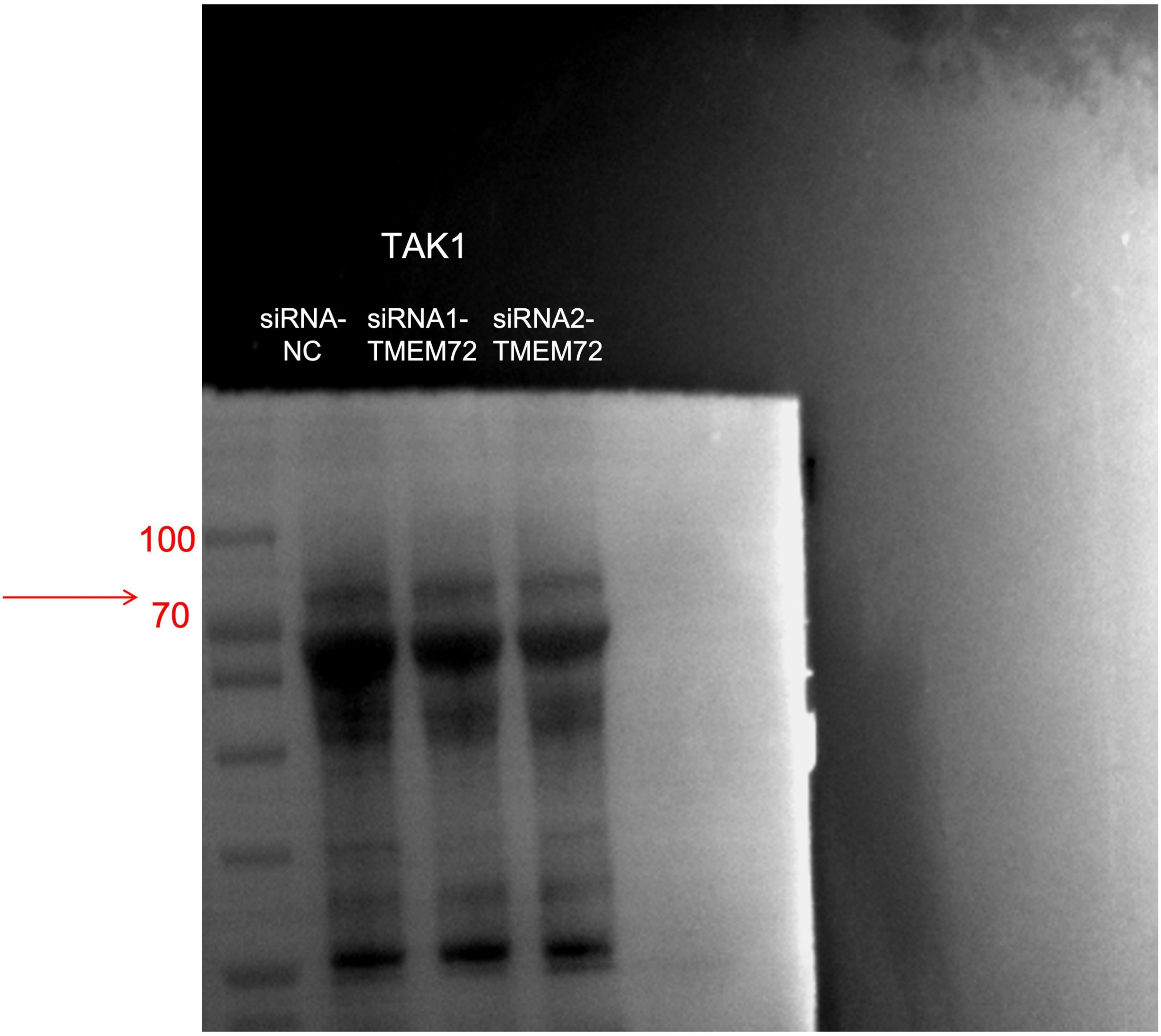

TAK1
siRNA-NC
siRNA1-TMEM72
siRNA2-TMEM72
100
70

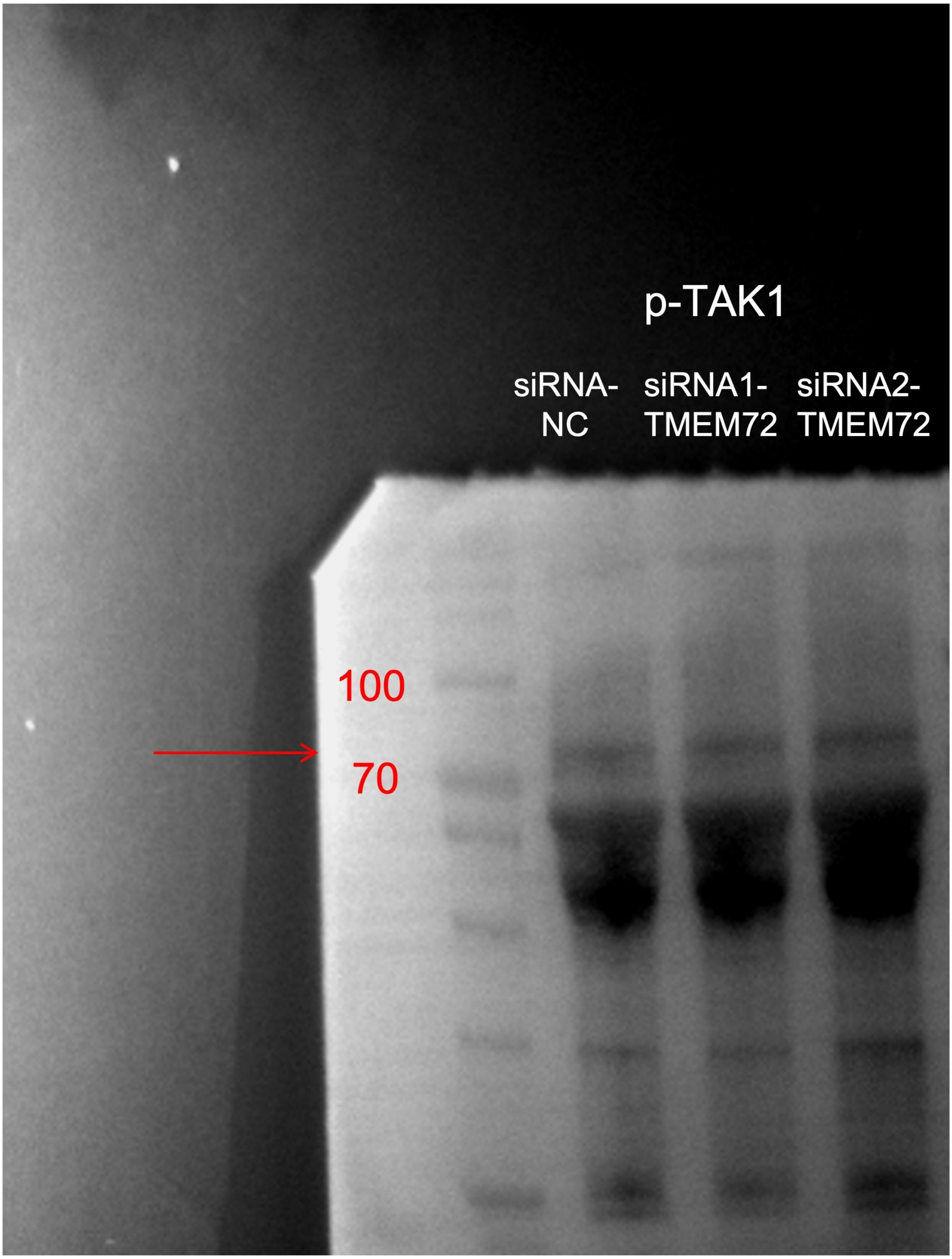

p-TAK1
siRNA-NC
siRNA1-TMEM72
siRNA2-TMEM72
100
70

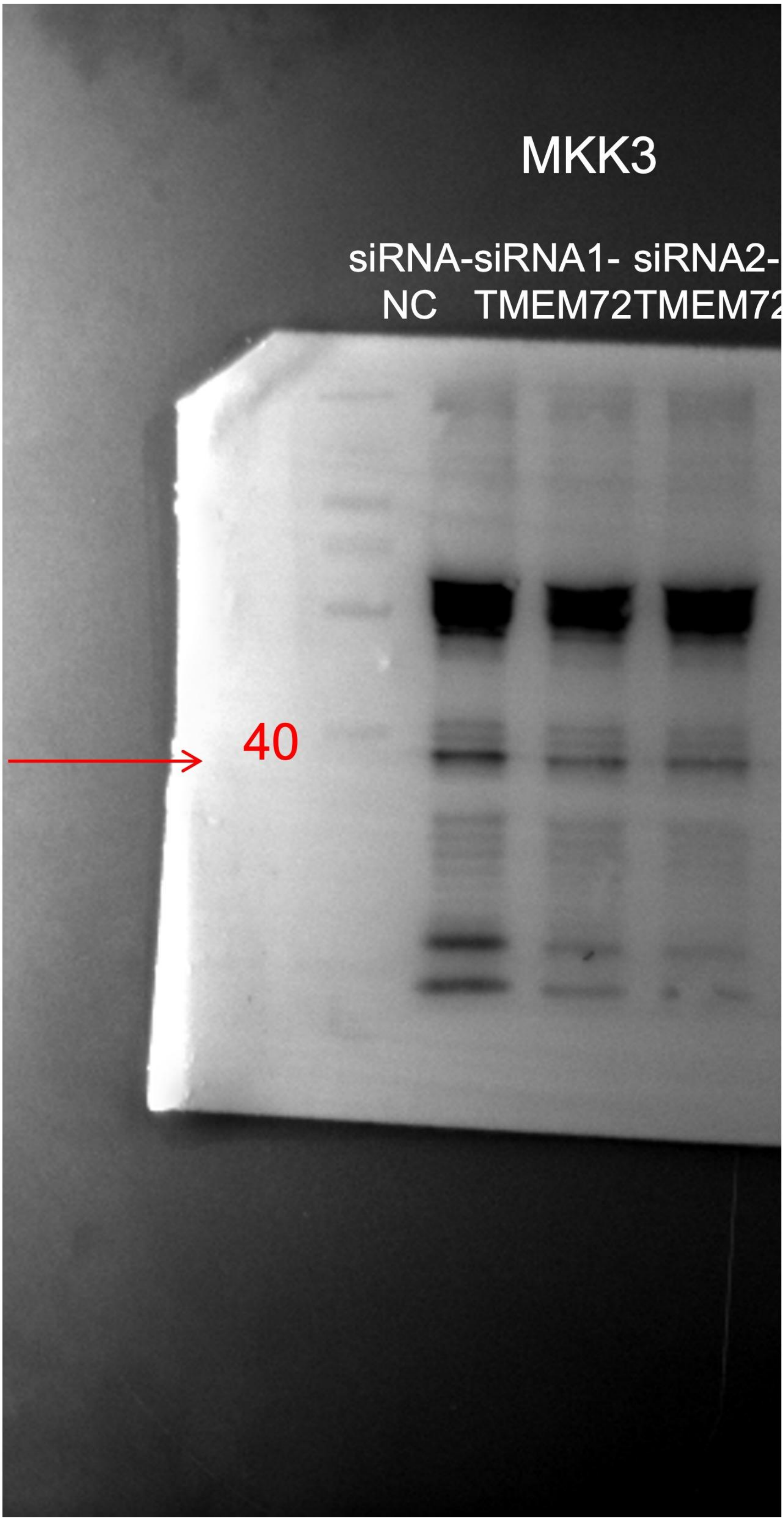
MKK3
siRNA- siRNA1- siRNA2-
NC TMEM72 TMEM7
40

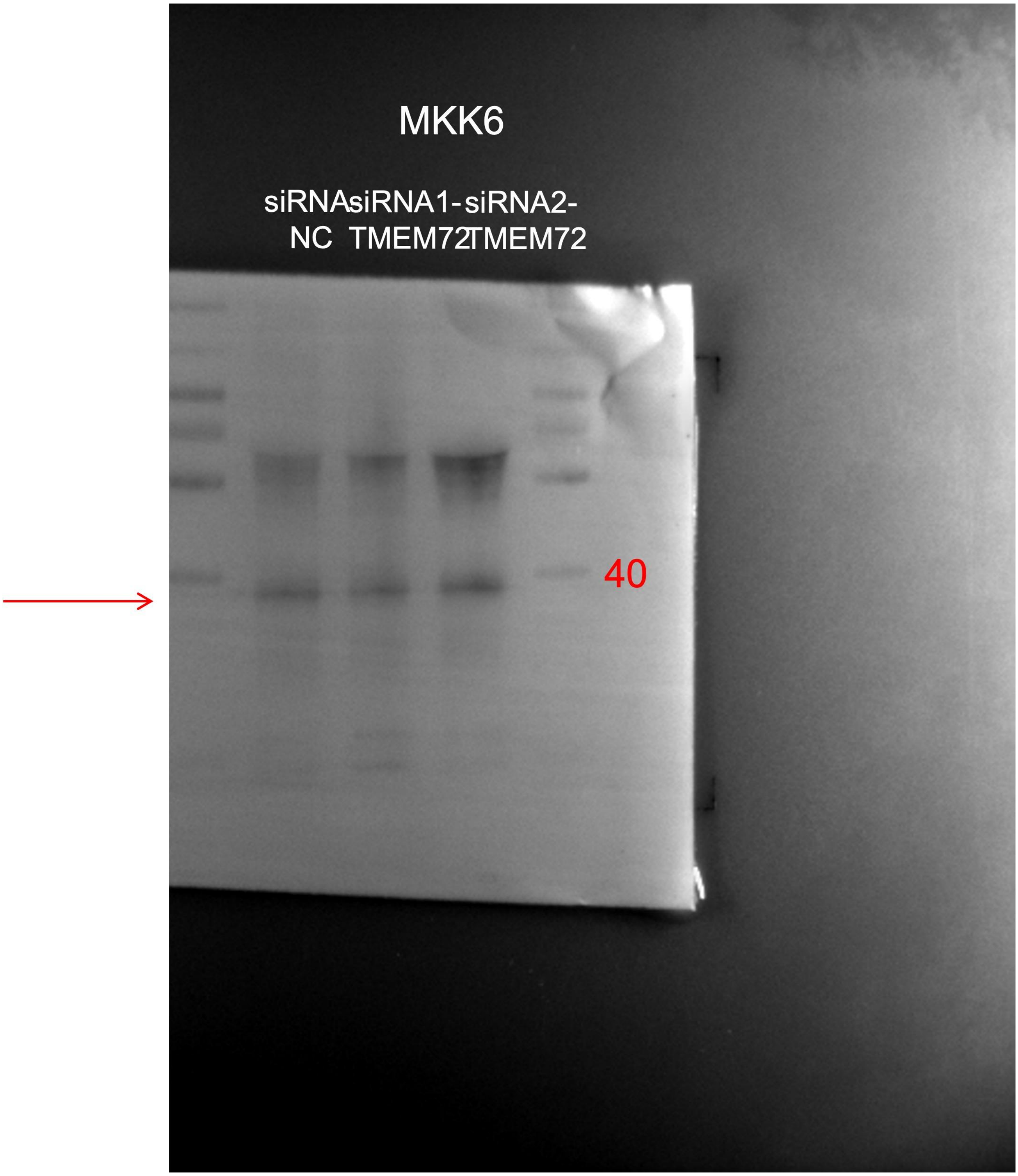
MKK6
siRNA-NC
siRNA1-TMEM72
siRNA2-TMEM72
40

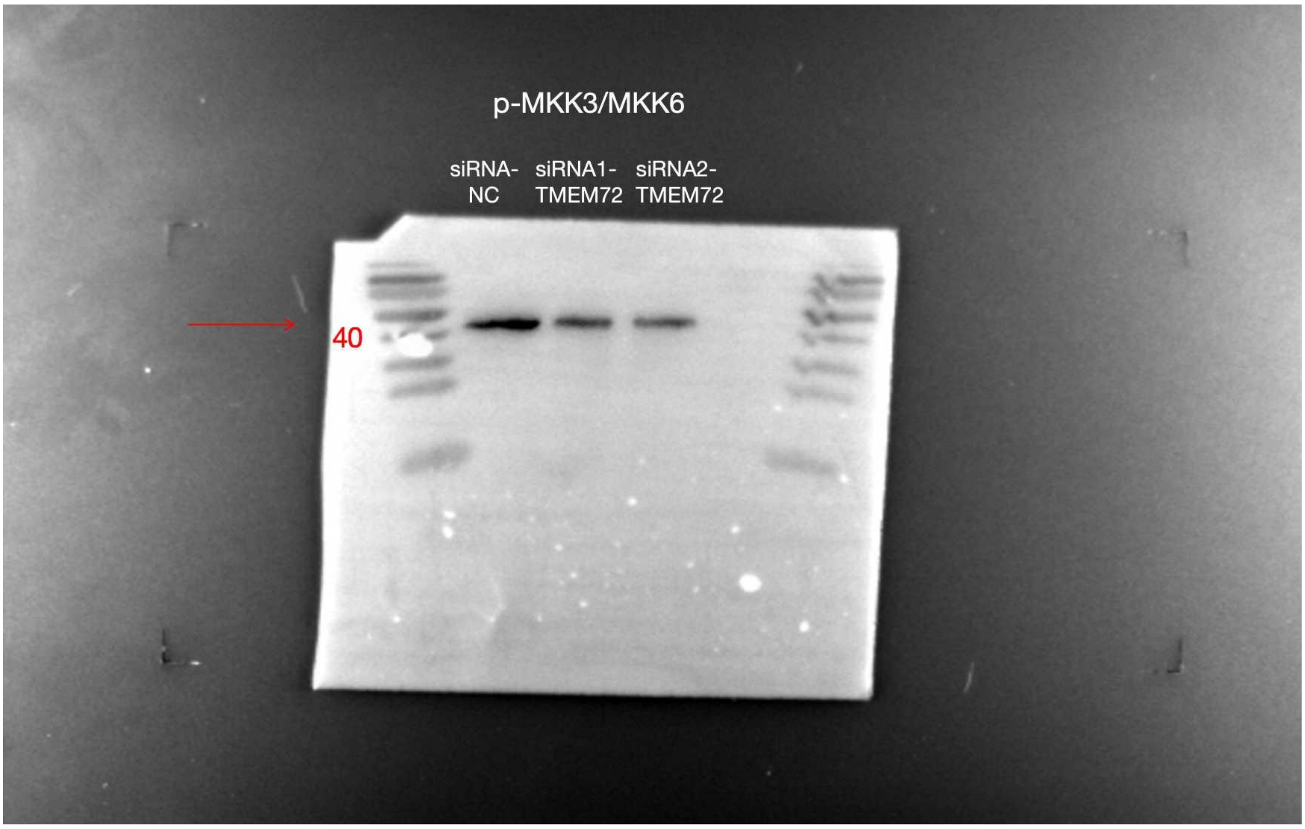
p-MKK3/MKK6
siRNA-NC
siRNA1-TMEM72
siRNA2-TMEM72
40

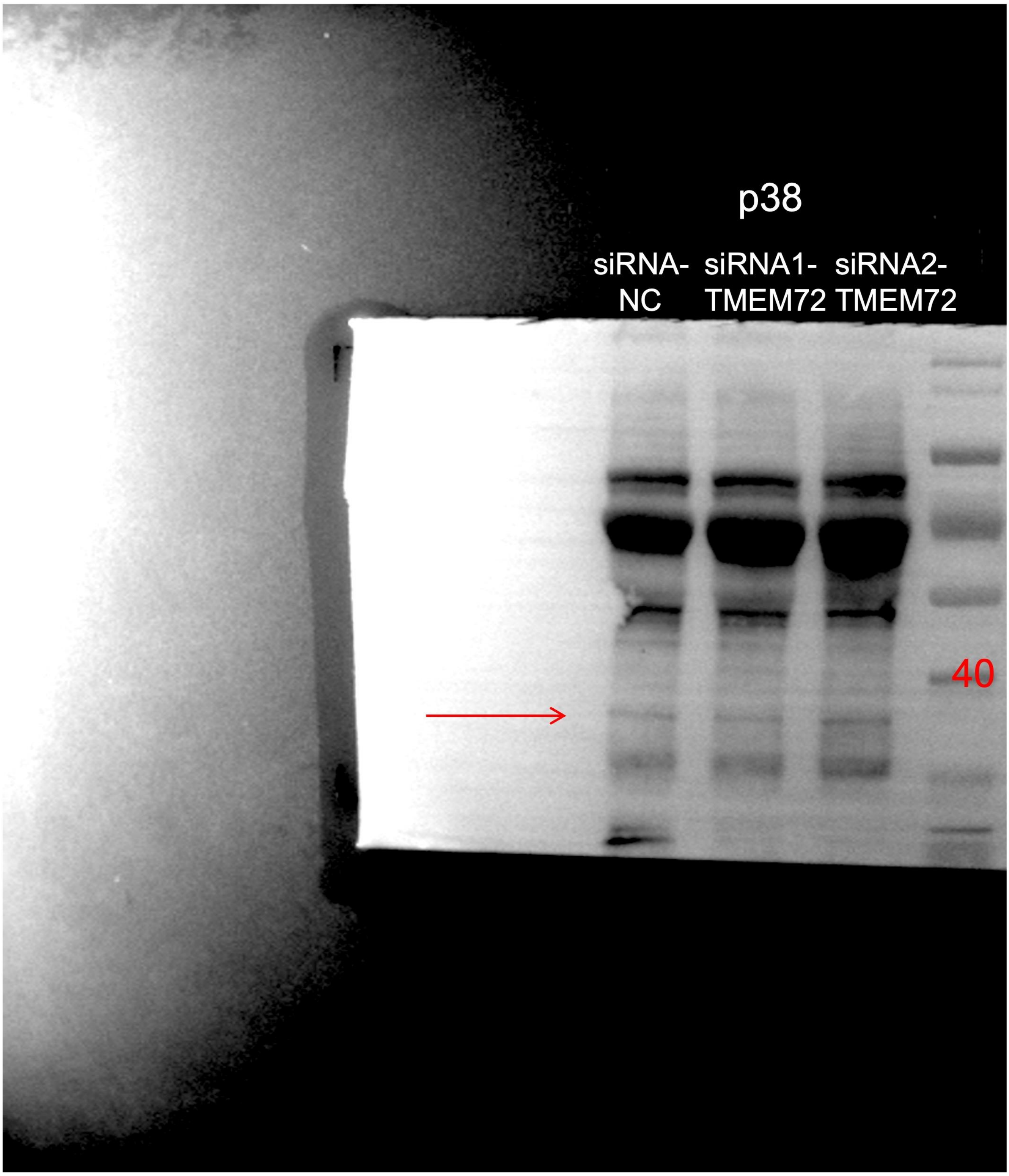
p38
siRNA-NC
siRNA1-TMEM72
siRNA2-TMEM72
40

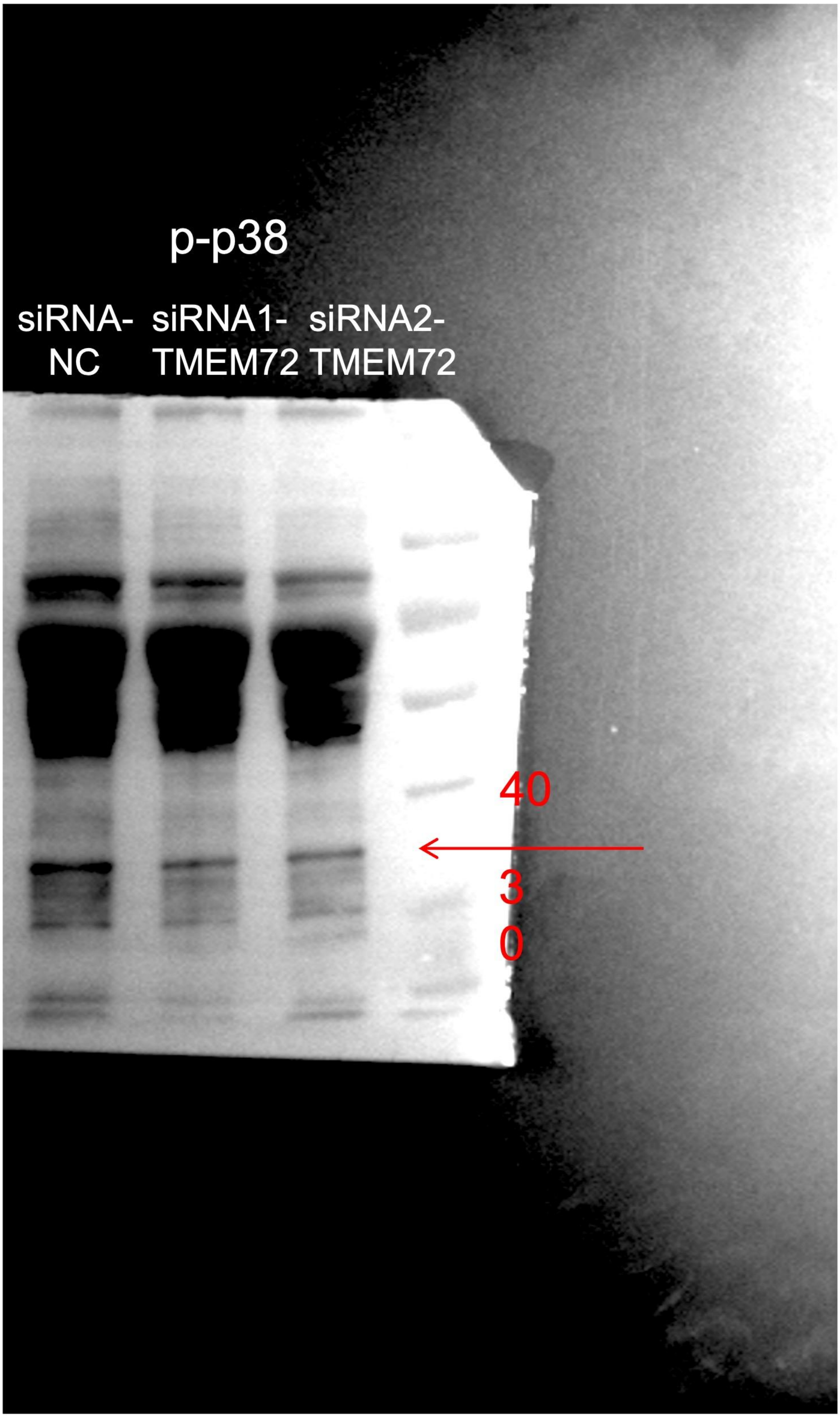
p-p38
siRNA-NC
siRNA1-TMEM72
siRNA2-TMEM72
40
30

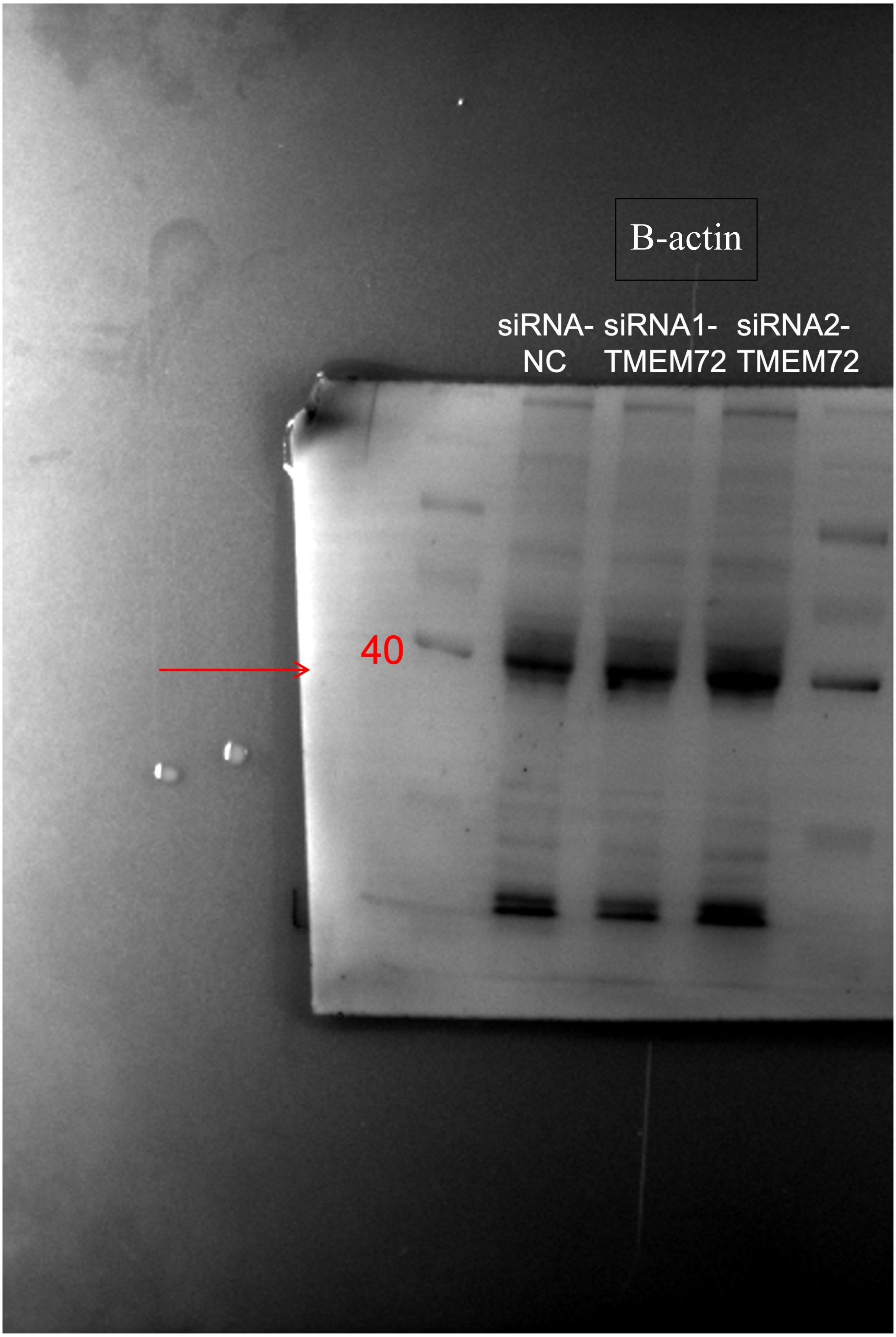
B-actin
siRNA-NC
siRNA1-TMEM72
siRNA2-TMEM72
40
